# A Pathogen-Inducible Rice NAC Transcription Factor ONAC096 Contributes to Immunity Against *Magnaprothe oryzae* and *Xanthomonas oryzae* pv. *oryzae* by Direct Binding to the Promoters of *OsRap2.6*, *OsWRKY62*, and *OsPAL1*

**DOI:** 10.3389/fpls.2021.802758

**Published:** 2021-12-10

**Authors:** Hui Wang, Yan Bi, Yizhou Gao, Yuqing Yan, Xi Yuan, Xiaohui Xiong, Jiajing Wang, Jiayu Liang, Dayong Li, Fengming Song

**Affiliations:** ^1^National Key Laboratory for Rice Biology, Institute of Biotechnology, Zhejiang University, Hangzhou, China; ^2^College of Chemistry and Life Science, Zhejiang Normal University, Jinhua, China

**Keywords:** NAC, *ONAC096*, rice immunity, *OsRap2.6*, *OsWRKY62*, *OsPAL1*

## Abstract

The rice NAC transcriptional factor family harbors 151 members, and some of them play important roles in rice immunity. Here, we report the function and molecular mechanism of a pathogen-inducible NAC transcription factor, ONAC096, in rice immunity against *Magnaprothe oryzae* and *Xanthomonas oryzae* pv. *oryzae*. Expression of *ONAC096* was induced by *M. oryzae* and by abscisic acid and methyl jasmonate. ONAC096 had the DNA binding ability to NAC recognition sequence and was found to be a nucleus-localized transcriptional activator whose activity depended on its C-terminal. CRISPR/Cas9-mediated knockout of *ONAC096* attenuated rice immunity against *M. oryzae* and *X. oryzae* pv. *oryzae* as well as suppressed chitin- and flg22-induced reactive oxygen species burst and expression of PTI marker genes *OsWRKY45* and *OsPAL4*; by contrast, overexpression of *ONAC096* enhanced rice immunity against these two pathogens and strengthened chitin- or flg22-induced PTI. RNA-seq transcriptomic profiling and qRT-PCR analysis identified a small set of defense and signaling genes that are putatively regulated by ONAC096, and further biochemical analysis validated that ONAC096 could directly bind to the promoters of *OsRap2.6*, *OsWRKY62*, and *OsPAL1*, three known defense and signaling genes that regulate rice immunity. ONAC096 interacts with ONAC066, which is a positive regulator of rice immunity. These results demonstrate that ONAC096 positively contributes to rice immunity against *M. oryzae* and *X. oryzae* pv. *oryzae* through direct binding to the promoters of downstream target genes including *OsRap2.6*, *OsWRKY62*, and *OsPAL1*.

## Introduction

To defend themselves against potential pathogens, plants have evolved a complicated and fine-tuned innate immune system, which consists of pattern-triggered immunity (PTI) and effector-triggered immunity (ETI) (Jones and Dangl, 2006; Boller and He, 2009; Zhang J. et al., 2020; Zhou and Zhang, 2020). PTI is the first layer of immune response that is initiated upon perception of conserved PAMP/MAMP/DAMP by cell surface-localized pattern recognition receptors (PRRs) (Boller and Felix, 2009; Kumar et al., 2021). ETI is the second layer of immune response that is stimulated upon direct or indirect recognition of pathogen-secreted effectors by intracellular resistance proteins (Jones and Dangl, 2006; Zhou and Zhang, 2020). In response to pathogen stimuli, transcription of numerous defense, and signaling genes is often reprogrammed in plants in an effective and timely manner to activate PTI and ETI (Seyfferth and Tsuda, 2014; Tsuda and Somssich, 2015; Windram and Denby, 2015; Birkenbihl et al., 2017; Mine et al., 2018; Chen et al., 2020; Yoo et al., 2020). Extensive studies have identified a large number of transcription factors (TFs), belonging to different families such as NAC, AP2/ERF, MYB, WRKY, bZIP, homeodomain, bHLH, NF-Y, and CAMTA, which play crucial roles in pathogen-induced transcriptional reprogramming of plant immune response (Alam et al., 2015; Huang P. Y. et al., 2016; Noman et al., 2017; He et al., 2018; Ng et al., 2018; Kim et al., 2020; Wani et al., 2021).

The NAC (NAM, ATAF1/2, and CUC2) family is one of the largest plant-specific TF families (Puranik et al., 2012). NAC proteins are characterized by a highly conserved N-terminal DNA-binding domain and a more diverse, intrinsically disordered C-terminal transcriptional regulatory domain (Olsen et al., 2005; Jensen et al., 2010; Puranik et al., 2012). NAC TFs have been shown to be involved in plant developmental processes, including leaf senescence, fruit ripening, secondary cell wall synthesis, lateral root development and seed size (Kim et al., 2016; Forlani et al., 2021; Kou et al., 2021; Singh et al., 2021). On the other hand, NAC TFs have also been demonstrated to regulate plant response to abiotic and biotic stress, including drought, salt, heat, cold, oxidative, metal toxicity, and pathogen attack (Puranik et al., 2012; Shao et al., 2015; Baillo et al., 2019; Yuan et al., 2019a,b; Bian et al., 2020; Diao et al., 2020; Ritonga et al., 2021; Warsi et al., 2021).

Rice genome encodes 151 NAC TFs (Ooka et al., 2003; Fang et al., 2008; Nuruzzaman et al., 2010) and the biological functions of some rice NAC TFs in growth, development and stress response have been elucidated (Chung et al., 2018; Yuan et al., 2019a,b; Zhang X. et al., 2020; Chang et al., 2021; Li et al., 2021). Particularly, nine rice NAC TFs, such as OsNAC6, OsNAC111, OsNAC60, ONAC122, ONAC131, OsNAC58, ONAC066, OsNAC4, and RIM1, have been implicated in rice immunity. OsNAC6 and OsNAC111 positively regulate rice immunity against *Magnaporthe oryzae*, probably through manipulating the expression of genes encoding for a cationic peroxidase, a DUF26 domain-containing protein, and pathogenesis-related proteins such as PR2 and PR8 (Nakashima et al., 2007; Yokotani et al., 2014). Overexpression of *OsNAC60* enhanced rice defense response to *M. oryzae*, accompanied with increased programmed cell death, greater ion leakage, more reactive oxygen species (ROS) accumulation and callose deposition, and up-regulation of defense genes (Wang et al., 2018). Silencing of *ONAC122* or *ONAC131* weakened rice resistance to *M. oryzae* and inhibited expression of several defense and signaling genes (Sun et al., 2013), while overexpression of *OsNAC58* increased rice resistance to *Xanthomonas oryzae* pv. *oryzae* (*Xoo*) (Park et al., 2017). ONAC066 positively contributes to rice immunity against *M. oryzae* and *Xoo* by directly binding to the promoters of the ABA biosynthetic genes *LIP9* and *NCED4* to suppress the ABA signaling pathway and binding to the promoters of *OsWRKY62* and a set of cytochrome P450 genes to activate defense response (Liu et al., 2018; Yuan et al., 2019b; Yuan X. et al., 2021). RIM1 represents a molecular link in jasmonic acid signaling and mutation in *RIM1* increased rice immunity against *Rice dwarf virus* (Yoshii et al., 2009, 2010). Overexpression of *OsNAC4* led to hypersensitive cell death, accompanying the loss of plasma membrane integrity and nuclear DNA fragmentation, suggesting that OsNAC4 is a key positive regulator of plant HR cell death (Kaneda et al., 2009). Therefore, the NAC TFs play significant roles in different aspects of rice immunity.

In our previous study, a number of *ONAC* genes that are responsive to biotic and abiotic stresses were identified through bioinformatics analysis of publicly available microarray data (Sun et al., 2015). Among these stress-responsive *ONAC* genes, *ONAC096* is closely related to *ONAC066*, which positively contributes to rice immunity against fungal and bacterial diseases (Liu et al., 2018; Yuan et al., 2019b; Yuan X. et al., 2021), and was significantly up-regulated in response to infection by *M. oryzae* and *Xoo* (Sun et al., 2015), implying the possible involvement of *ONAC096* in rice immunity. In the present study, a detailed functional analysis of *ONAC096* in rice immunity was carried out by overexpression and CRISPR/Cas9-mediated knockout of *ONAC096* in transgenic rice and using a T-DNA insertional mutant. Results from this study revealed that ONAC096 positively contributes to rice immunity against *M. oryzae* and *Xoo* through direct binding to the promoters of downstream target genes including *OsRap2.6, OsWRKY62*, and *OsPAL1*.

## Materials and Methods

### Plant Materials and Growth Conditions

Rice (*Oryza sativa* L.) cv. Zhonghua 11 (ZH11) were used for the analysis of gene expression after hormone treatment and for generation of *ONAC096* transgenic lines while a pair of near isogenic lines H8S and H8R was used for the analysis of gene expression in compatible and incompatible interactions with *M. oryzae*. Seeds were pre-germinated for 3 days and were then planted into a soil mix (clay: soil = 3:1). Rice plants were grown in a growth room at 26∼28°C with 60% relative humidity (RH) and a 14 h light/10 h dark cycle, as previously described (Hong et al., 2017).

### Hormone Treatment and Pathogen Inoculation

Hormone treatment was performed as previously described (Sun et al., 2013). Briefly, plants at 3∼4-leaf stage were foliar sprayed with 100 μM abscisic acid (ABA), 100 μM methyl jasmonate (MeJA) in 0.1% ethanol and 0.02% Tween-20 or with equal volume of 0.1% ethanol and 0.02% Tween-20 solution as mock controls. For analysis of *ONAC096* expression in H8S/H8R-*M. oryzae* interactions, blast fungus strain RB22 was grown on CM medium at 25°C for 10 days and spores were collected to prepare inoculum as previously described (Hong et al., 2017; Yuan et al., 2019b; Yuan X. et al., 2021). Plants of H8S/H8R at 3∼4-leaf stage were foliar sprayed with 2× 10^5^ spores/mL spore suspension containing 0.05% Tween-20 or with equal volume of 0.05% Tween-20 solution as mock controls (Li et al., 2011; Hong et al., 2017; Yuan et al., 2019b; Yuan X. et al., 2021). The inoculated plants were kept at 25°C in dark for 24 h with 100% RH, and then moved to the growth room with the same condition. Leaf samples were collected at indicated time points after hormone treatment or pathogen inoculation, frozen in liquid nitrogen, and stored at -80°C until use.

### Subcellular Localization Assay

*ONAC096* coding sequence was amplified with gene-specific primers (Supplementary Table 1) and cloned into pCAMBIA1300s, generating pCAMBIA1300s-ONAC096-GFP. Agrobacteria carrying pCAMBIA1300s-ONAC096-GFP or pCAMBIA1300s-GFP were infiltrated into leaves of *Nicotiana benthamiana* plants expressing a known nucleus-localized marker protein RFP-H2B (Chakrabarty et al., 2007) as previously described (Yuan et al., 2019a). At 48 h after agroinfiltration, GFP fluorescence was observed and imaged under a Zeiss LSM780 confocal laser scanning microscope (Zeiss, Oberkochen, Germany).

### Transactivation Activity Assay

Transactivation activity of ONAC096 was determined by yeast one-hybrid (Y1H) assay as previously described (Hong et al., 2016; Yuan et al., 2019a). Coding sequence and truncated sequences of *ONAC096* were amplified with gene-specific primers (Supplementary Table 1) and inserted into pGBKT7, separately. The recombinant plasmids, pGBKT7 (a negative control) or pGBKT7-ONAC022 (a positive control) (Hong et al., 2016) were introduced into yeast strain AH109. Transactivation activity of the fused ONAC096 and its truncated mutants was assessed by growth performance and appearance of blue pigment after addition of 5-bromo-4-chloro-3-indolyl-α-D-galactopyranoside (X-α-gal) (TaKaRa, Dalian, China) on SD/-Trp-His medium containing 3-amino-1, 2, 4-triazole (3-AT) (Clontech, Mountain View, CA, United States).

### DNA Binding Activity Assay

The DNA binding activity of ONAC096 to the *NACRS* element (5′-CCATGTGGAGCACGGAGCACGA-3′) was analyzed by Y1H assay as previously described (Yuan et al., 2019a). The coding sequence of *ONAC096* was amplified with gene-specific primers (Supplementary Table 1) and cloned into pGADT7-Rec2, yielding pGADT7-Rec2-ONAC096 while 3 × *NACRS* in tandem was inserted into reporter vector pHis2, yielding pHis2-3 × *NACRS* (Yuan et al., 2019a). The sequence or fragments in the promoters (1.5 Kb upstream from ATG) of candidate target genes were amplified and cloned into pHis2. The fused pGADT7-Rec2-ONAC096 or pGADT7-Rec2 were co-transformed with each of the recombinant pHis2 constructs or empty vector pHis2 into yeast strain Y187. The DNA binding activity of ONAC096 and DNA-protein interaction were evaluated by the growth status of yeast cells on SD/-Trp-Leu-His medium containing 3-AT.

The binding of GST-ONAC096 to *cis*-elements in the *OsRap2.6* promoter was determined by Electrophoretic Mobility Shift Assay (EMSA) as previously described (Hong et al., 2016). The coding sequence of *ONAC096* was amplified using gene-specific primers (Supplementary Table 1) and cloned into pGEX-4T-3, generating fusion plasmid GST-ONAC096, which was introduced into the *E. coli* strain Rosetta DE3. Expression of GST-ONAC096 protein was induced by an addition of 1 mM isopropyl-D-thiogalactoside (IPTG) at 18°C overnight and was purified using glutathione resin column (Genscript, Shanghai, China) according to the manufacturer’s protocol. A 36 bp wP2 fragment (AAAACACGTATACAGCCAAAATACGTTACACGGTGG, core sequence underlined) and a 28 bp wP4 fragment (GTAAG GTGGATCCACGGGTCAGGCATGG, core sequence underlined) in the *OsRap2.6* promoter as well as mutated fragments mP2 (AAAAAAAATATACAGCCAAAATACGTTA AAAAGTGG, mutated nucleotides underlined) and mP4 (GTAAGGTGGATCAAAAGGTCAGGCATGG, mutated nucleotides underlined) were synthesized and labeled with biotin. EMSA was performed as previously described (Yuan et al., 2019a) using LightShift Chemiluminescent EMSA Kit (Thermo Fisher Scientific, Waltham, MA, United States). Briefly, binding reactions (10 μL) contained 1 μL 10 × binding buffer, 2 μg GST-ONAC096 protein or GST protein (a negative control) and 1 μL biotin-labeled wP2/wP4 or mP2/mP4 probe. For the competitive reactions, unlabeled wP2/wP4 or mP2/mP4 probe was added in excess of 100 times. The reactions were incubated at 28°C for 20 min, separated on 6% native PAGE gels and transferred onto Amersham Hybond-N^+^ nylon transfer membrane (GE Healthcare, Buckinghamshire, United Kingdom). Signals from the biotin-labeled probes were detected using a Chemiluminescent Biotin-labeled Nucleic Acid Detection Kit (Beyotime Biotechnology, Haimen, China) according to the manufacturer’s recommendations.

### Bimolecular Fluorescence Complementation Assay

Bimolecular fluorescence complementation assay for determining the ONAC096-ONAC096, ONAC066-ONAC066, and ONAC096-ONAC066 interactions was carried out as previously described (Liu et al., 2019). Coding sequences of *ONAC066* and *ONAC096* were amplified with gene-specific primers (Supplementary Table 1) and inserted into p2YC, yielding p2YC-ONAC066 and p2YC-ONAC096, or cloned into p2YN, generating p2YN-ONAC066 and p2YN-ONAC096. Agrobacteria harboring different indicated pairs of fused or empty plasmids were infiltrated into leaves of *N. benthamiana* plants expressing a red nuclear marker protein RFP-H2B (Chakrabarty et al., 2007) as previously described (Liu et al., 2019). At 48 h after agroinfiltration, YFP and RFP signals were detected and photographed under a Zeiss LSM780 confocal laser scanning microscope (Zeiss, Oberkochen, Germany).

### Characterization of Transgenic and T-DNA Insertional Lines

The constructed plasmids pCAMBIA1300s-ONAC096-GFP and pYLCRISPR/Cas9*-*ONAC096 were introduced into ZH11 (Wuhan Biorun Biotechnology Co., Wuhan, China), respectively, using the *Agrobacterium tumefaciens*-mediated transformation method to generate *ONAC096*-OE and *ONAC096*-CP lines. For the characterization of *ONAC096*-OE plants, homozygous single-copy transgenic lines were screened as previously described (Huang L. et al., 2016; Yuan et al., 2019a). Briefly, transgenic lines with single-copy transgene were selected from T2 generation of the transgenic lines with 3:1 segregation for hygromycin (Hgr)-resistant: susceptible phenotype on 1/2 MS medium containing 50 μg/L Hgr. To confirm these lines, genomic DNA was extracted using CTAB method and ∼50 μg genomic DNA was digested completely with *Kpn*I. After separation on a 0.8% agarose gel, DNA was transferred onto an Amersham Hybond-N^+^ nylon membrane (GE Healthcare, Buckinghamshire, United Kingdom), hybridized with a 289 bp *Hpt*II probe labeled with DIG and the signal was detected using a DIG High Prime DNA Labeling and Detection Kit I (Roche Diagnostics, Shanghai, China) according to the manufacturer’s instruction. The single-copy lines with 100% Hgr-resistant phenotype in T3 generation were chosen as homozygous single-copy transgenic lines. For the characterization of *ONAC096*-CP lines, homozygous transgenic lines were identified as previously described (Ma et al., 2015; Zeng et al., 2020). Briefly, the regions flanking the two designed targets in T1 generation of transgenic lines for Hgr-resistant phenotype were amplified with gene-specific primers (Supplementary Table 1) and the PCR products were sequenced to screen the homozygous *ONAC096*-CP lines. A T-DNA insertional line for *ONAC096* (1B-02928.R) in *japonica* cv. Dongjin (DJ) background was obtained from POSTECH RISD (Rice T-DNA Insertion Sequence Database) (Jeon et al., 2000; An et al., 2003). Homozygous plants were identified by PCR-based genotyping with gene-specific and T-DNA-specific (2715LB) primers (Supplementary Table 1; Yuan et al., 2019b; Yuan X. et al., 2021). Leaf samples were harvested from homozygous *ONAC096*-OE, *ONAC096*-CP and *onac096* plants for analysis of *ONAC096* transcript level and ONAC096-GFP protein level. The *ONAC096*-CP-14, *ONAC096*-CP-37, *ONAC096*-OE-12, *ONAC096*-OE-15, and *onac096* lines were used for experiments.

### Disease Assays

Foliar spraying and punch inoculation methods were used for blast disease assay. Foliar spraying inoculation was carried out as described above. Lesion numbers were counted and leaf samples were collected at 5 days post inoculation (dpi). For punch inoculation, detached leaves were inoculated by dropping 5 μL spore suspension (5 × 10^5^ spores/mL in 0.05% Tween-20) on leaf surface and were kept in dark at 25°C under 100% RH for 24 h before returning to the growth room (Yuan et al., 2019b; Yuan X. et al., 2021). Lesion length was evaluated and leaf samples were collected at 5 dpi. For determination of *in planta* fungal growth, qRT-PCR was performed to analyze the genomic DNA level of *M. oryzae MoPot* and rice *ubiquitin* (*OsUbq*) genes using gene-specific primers (Supplementary Table 1). Relative fungal growth was presented as folds of the genomic fungal *MoPot* gene level to rice *OsUbq* gene level (Park et al., 2012; Yuan et al., 2019b; Yuan X. et al., 2021).

Leaf-clipping method was used for blight disease assay (Hong et al., 2017). Briefly, *Xoo* strain PXO89 was grown on NA agar medium at 28°C for 24 h and re-suspended in distilled water at OD_600_ = 0.8. Plants at booting stage were inoculated with bacterial suspension using the leaf-clipping method and were then kept in a greenhouse at 26∼28°C with natural sunlight. Disease phenotype and lesion area were estimated at 14 dpi. For measurement of bacterial growth, leaf samples were surface sterilized in 70% ethanol for 10 s, homogenized in 200 μL distilled water and serially diluted to different concentrations. An aliquot of 100 μL of the proper dilutions were plated on NA agar medium. After incubation at 28°C for 24 h, the colony-forming units (CFU) were counted and the bacterial growth was presented as log_10_ (CFU/cm^2^).

### Reactive Oxygen Species Assay

Reactive Oxygen Species assays were conducted as previously described (Liu et al., 2017, 2019; Wang et al., 2019). Briefly, leaf discs (0.25 cm^2^) were pre-incubated overnight in a 96-well plate with sterilized ddH_2_O, and the reactions were initiated by adding 200 μM luminol (Sigma-Aldrich, Saint Louis, MO, United States), 20 μg/mL horseradish peroxidase (Sigma-Aldrich, Saint Louis, MO, United States), and 100 nM flg22 (GenScript, Nanjing, China), 1 μM chitin (IsoSep, Tullinge, Sweden), or ddH_2_O as controls. Chemiluminescent signal was immediately measured at a 1 min interval over a period of 30 min using a Synergy HT plate reader (Biotek Instruments, Inc., Winooski, VT, United States).

### qRT-PCR Assay

Total RNA was extracted using RNA Isolater reagent (Vazyme, Nanjing, China) according to the manufacturer’s recommendations. The isolated RNA was treated with genome DNA wiper mix (Vazyme, Nanjing, China) to remove the remaining genomic DNA and then reverse-transcribed into first-strand cDNA using the reverse transcription system (Vazyme, Nanjing, China). Each qRT-PCR reaction (20 μL) contained 10 μL 2 × AceQ qPCR SYBR Green Master Mix (Vazyme, Nanjing, China), 0.1 μg cDNA and 0.25 μM each of gene-specific primer (Supplementary Table 1) and was run on a CFX96 real-time PCR system (Bio-Rad, Hercules, CA, United States). PCR conditions were as follow: 95°C for 5 min, 40 cycles of 95°C for 10 s, 60°C for 15 s and 72°C for 30 s. A rice *18s rRNA* gene was used as an internal control to normalize the qRT-PCR data (Yuan et al., 2019a,b; Yuan X. et al., 2021) and relative gene expression was calculated using the 2^–ΔΔCT^ method.

### Chromatin Immuno-Precipitation and ChIP-PCR

ChIP-PCR assay was performed as previously described (Yuan et al., 2019a,b; Yuan X. et al., 2021). Briefly, ∼4 g leaves from *ONAC096*-OE12 plants were harvested and immediately crosslinked in 1% formaldehyde by vacuum infiltration for 30 min. After an addition of 125 mM glycine to stop the cross-linking, the chromatin DNA was extracted and fragmented to 200∼500 bp by sonication (Bioruptor Plus, Diagenode, Belgium). The fragmented DNA was pre-cleaned with Pierce Protein A/G Magnetic Beads (Thermo Fisher Scientific, Waltham, MA, United States) and an aliquot (10%) of the chromatin DNA was used as an input control. The remaining pre-cleaned chromatin DNA was incubated overnight with GFP antibody (Sigma-Aldrich, Saint Louis, MO, United States) or with pre-immune (Pre) serum (GenScript, Nanjing, China) (a negative control). After reversal of the cross-links, the immunoprecipitated DNA and input DNA were extracted by phenol/chloroform extraction. PCR analysis of ChIP DNA and input DNA were carried out using gene-specific primers (Supplementary Table 1).

### RNA-Seq Analysis

Four-week-old *ONAC096*-CP, *ONAC096*-OE, and WT plants were inoculated by foliar spraying with *M. oryzae* spore suspension (2 × 10^5^ spores/mL) or mock-inoculated as controls. At 24 h after inoculation, leaf samples were collected from three biological replicates (∼6 plants each) for RNA-seq. Solexa sequencing on the Hiseq 2500 platform (Illumina) and data analysis were carried out by BioMarker Technologies (Beijing, China). Raw sequence reads were cleaned by removing low quality tags, adaptor sequences, contaminant DNA, and PCR duplicates. All clean reads were mapped to reference genome (GCF_001433935.1_ IRGSP_1.0) using TopHat 2 tools software (v2.0.12) (Kim et al., 2013). Gene expression levels were obtained by estimating the FPKM (Fragments Per Kilobase per Million mapped reads) values using RSEM program (Li and Dewey, 2011). Differential expression analysis was performed with DESeq R package 1.10.1 (Anders and Huber, 2010). Differentially expressed genes (DEGs) were defined by expression change ≥ 1.5-fold with *P* value < 0.05. Gene Ontology (GO) enrichment analysis of the DEGs was implemented by the GOseq R packages based Wallenius non-central hyper-geometric distribution (Young et al., 2010).

### Experiment Design and Statistical Analysis

All experiments were performed in triplicate and data are shown as mean ± standard errors (SE) from three independent experiments. Data were subjected to statistical analysis according to the Student’s *t* test and the probability value of *p < 0.05 or ^**^p < 0.01 was considered as significant difference.

## Results

### *ONAC096* Is a Pathogen- and Defense Hormone-Inducible *ONAC* Gene

The *ONAC096* gene (LOC_Os07g04560) comprises a 1032 bp open reading frame encoding a 343 amino acid (aa) protein with a typical conserved NAC domain at N-terminus, which can be divided into five subdomains, namely A, B, C, D, and E (Ooka et al., 2003; Supplementary Figure 1A). Phylogenetic tree analysis indicates that ONAC096 belongs to Phylogeny Group IV (Fang et al., 2008), and is closely related to ONAC066, ONAC140, and ANAC042/AtJUB1 (Supplementary Figure 1B). Bioinformatics analysis at PlantCARE identifies several stress response-related *cis*-elements including four ABREs elements, four CGTCA motifs, four TGACG motifs, one TATC box, and one TCA element in the *ONAC096* promoter region (1.5 Kb upstream of ATG) (Supplementary Figure 1C), implying that *ONAC096* may be a stress-responsive *ONAC* gene in rice.

**FIGURE 1:**
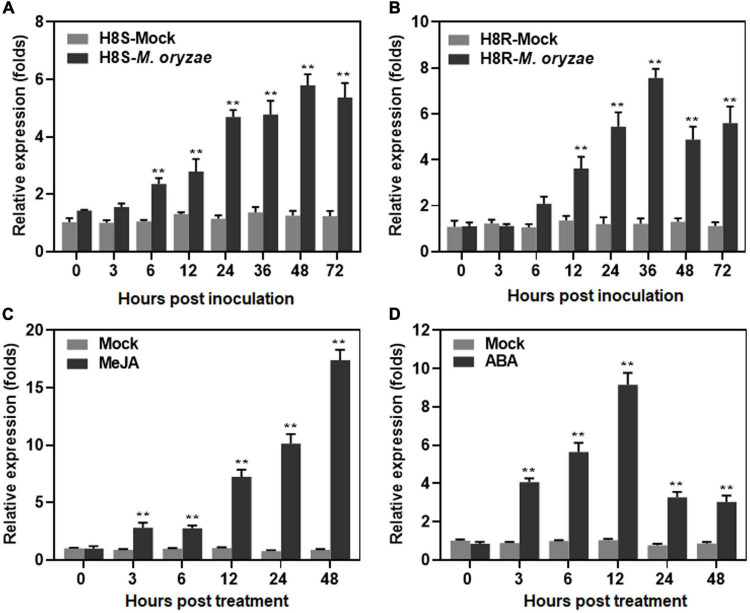
Responsiveness of *ONAC096* to *M. oryzae* and defense signaling hormones. **(A,B)** Expression changes of *ONAC096* in compatible **(A)** and incompatible **(B)** interactions between rice and *M. oryzae*. Three-week-old rice seedlings of H8S (compatible interaction) and H8R (incompatible interaction) were inoculated by foliar spraying with spore suspension of *M. oryzae* (2 × 10^5^ spores/mL) or equal water as mock controls. **(C,D)** Expression changes of *ONAC096* after treatment with defense signaling hormones ABA **(C)** or JA **(D)**. Two-week-old rice seedlings of cv. ZH11 were treated by foliar spraying with 100 μM ABA, 100 μM JA or equal water as mock controls. Leaf samples were collected at indicated time points after *M. oryzae* inoculation **(A,B)** or hormone treatment **(C,D)**. Relative expression levels of *ONAC096* as compared to those of the *18S rRNA* gene are presented as the means ± SE from three independent experiments. Asterisks indicate significant difference (*p* < 0.01, Student’s *t* test) in comparison to mock inoculation **(A,B)** or mock treatment **(C,D)** at each time point.

To explore the involvement of *ONAC096* in biotic stress response, the expression changes of *ONAC096* were analyzed in compatible and incompatible interactions between rice and *M. oryzae* and in response to defense signaling hormones ABA or JA. The expression of *ONAC096* was significantly and rapidly up-regulated in H8S (compatible interaction) and H8R (incompatible interaction) within 72 h after *M. oryzae* infection. In *M. oryzae*-inoculated H8S plants, expression of *ONAC096* started to increase at 3 h post inoculation (hpi) and increased gradually over a period of 72 h, with a peak at 48 hpi, leading to 4.6-fold higher than that in mock-inoculated plants (Figure 1A). Similarly, in *M. oryzae*-inoculated H8R plants, expression of *ONAC096* increased at 6 hpi and peaked at 36 hpi, resulting in a 6.2-fold, higher than that in mock-inoculated plants (Figure 1B). A high level of *ONAC096* expression was detected in JA-treated plants, showing 7.1∼20.2-fold higher than those in untreated control plants during 12∼48 h (Figure 1C). Moreover, significant induction of *ONAC096* expression was observed in ABA-treated plants at 6 and 12 h after treatment, giving 5.7- and 9.0-fold higher than those in untreated control plants (Figure 1D). These results indicate that *ONAC096* is a pathogen- and defense hormone-inducible *ONAC* gene.

### *ONAC096* Harbors DNA Binding for Transactivation Activities in Plant Nuclei

**FIGURE 2:**
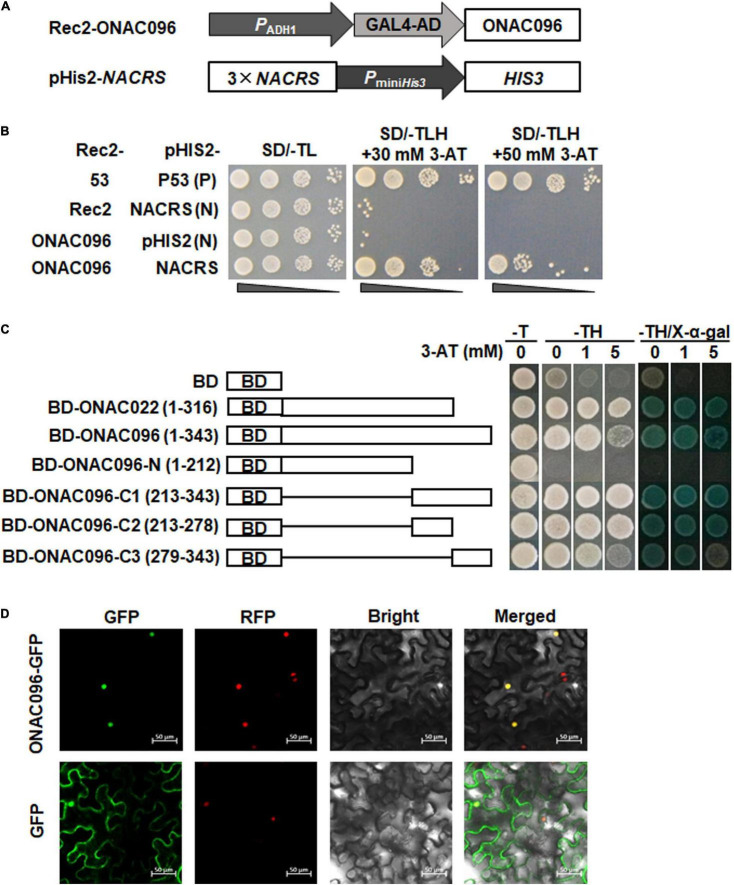
DNA binding and transactivation activities and subcellular localization of ONAC096 protein. **(A)** Schematic diagram of plasmid constructs used in ONAC096-*NACRS* binding assay. **(B)** ONAC096 binds to *NACRS* in yeast cells. Yeast cells co-transformed with indicated pairs of Rec2 and pHis2 vectors were incubated on SD/-Trp-Leu plates at 30°C for 3 days and were dropped by a series of 10-fold dilutions on plates of SD/-Trp-Leu, SD/-Trp-Leu-His/30 mM 3-AT, and SD/-Trp-Leu-His/50 mM 3-AT. Transformants harboring Rec2-53 and pHis2-P53 were used as positive controls whereas transformants carrying Rec2 and pHis2-*NACRS* or carrying Rec2-ONAC096 and pHis2 were used as negative controls. **(C)** ONAC096 has transactivation activity in yeast cells. Full sequence and truncated mutants of *ONAC096* were fused to GAL4 DNA-binding domain in pGBKT7 vector. Yeast cells harboring each of the different truncated and mutated constructs, pGBKT7 empty vector (a negative control) or pGBKT7-ONAC022 (a positive control) were grown on SD/-Trp or SD/-Trp-His plates with different concentration of 3-AT at 30°C for 3 days. Transactivation activity was examined by the appearance of blue color after addition of X-α-gal in SD/-Trp-His/3-AT plates for 1 day. BD, pGBKT7 vector with the GAL4 DNA-binding domain. **(D)** ONAC096 is localized in nucleus. Agrobacteria carrying pCAMBIA1300-ONAC096-GFP or pCAMBIA1300-GFP were infiltrated into leaves of *N. benthamiana* plants expressing a known nucleus-localized marker protein RFP-H2B. At 48 h after agroinfiltration, GFP signals were visualized under a confocal laser scanning microscope in dark field for green fluorescence (*left*), red fluorescence (*middle left*), white field for cell morphology (*middle right*) and in combination (*right*), respectively. Scale bar = 50 μm. Experiments in panels **(B–D)** were repeated for three times with similar results and results from one representative experiment are shown.

To determine whether ONAC096 possess DNA binding activity, the binding capacity of ONAC096 to *NACRS*, a canonical NAC core binding sequence (Tran et al., 2004), was examined using Y1H assay. The tandem repeated 3 × *NACRS* into pHis2 vector (Figure 2A) was co-transformed with Rec2-ONAC096 or pGADT7-Rec2 into yeast cells. All yeast cells grew well in SD/-Trp-Leu medium (Figure 2B). Whereas only yeast cells carrying Rec2-ONAC096 and pHis2-3 × *NACRS* grew normally on SD/-Trp-Leu-His medium with 30 mM or 50 mM 3-AT, yeast cells co-transformed with pGADT7-Rec2 and pHis2-3 × *NACRS* or Rec2-ONAC096, and pHis2 did not grow (Figure 2B). These results indicate that ONAC096 can bind to the NAC binding sequence, *NACRS*, and thus has DNA binding activity.

To explore whether ONAC096 has transcriptional activator activity, the entire ONAC096 (1-343 aa) and a series of truncated variants including ONAC096-N (1-212 aa), ONAC096-C1 (213-343 aa), ONAC096-C2 (213-278 aa), and ONAC096-C3 (279-343 aa) were each fused to GAL4 DNA-binding domain. All yeast cells grew well on SD/-Trp medium (Figure 2C, *left*). Yeast cells harboring pGBKT7-ONAC022 (a positive control) (Hong et al., 2016), pGBKT7-ONAC096, or variants pGBKT7-ONAC096-C1, pGBKT7-ONAC096-C2, or pGBKT7-ONAC096-C3 grow normally and show β-galactosidase activity while yeast cells harboring pGBKT7-ONAC096-N failed to grow on SD/-Trp-His medium with different concentration of 3-AT (Figure 2C, *right*). These results indicate that ONAC096 has transactivation activity and the C-terminus is critical for its transactivation activity.

To examine the subcellular localization of ONAC096, agrobacteria carrying ONAC096-GFP or GFP were infiltrated into leaves of *N. benthamiana* plants expressing a red nuclear marker RFP-H2B protein (Chakrabarty et al., 2007). The ONAC096-GFP protein was solely and clearly localized to the nucleus, which was co-localized with the known nucleus marker RFP-H2B (Figure 2D, *top*). In contrast, GFP alone distributed ubiquitously throughout the cell without specific compartmental localization (Figure 2D, *bottom*). These results indicate that ONAC096 is a nucleus-localized protein.

### *ONAC096* Positively Regulates Rice Immunity Against Both *Magnaprothe oryzae* and *Xoo* Infection

To investigate the function of *ONAC096*, two transgenic rice lines with CRISPR/Cas9-mediated knockout of *ONAC096*, *ONAC096*-CP-14 and -CP-37, were obtained (Supplementary Figures 2A,B), and the transcript level of *ONAC096* in CP-14 and CP-37 lines was estimated to be 7.5 and 4.7% of that in wild type (WT) plants (Supplementary Figure 2C). Meanwhile, three independent *ONAC096*-overexpressing lines, *ONAC096*-OE-12, -OE-15, and -OE-16, were generated (Supplementary Figures 3A,B), and a 4.5∼9.4-fold higher level of *ONAC096* transcript and a ∼64.8 kDa ONAC096-GFP protein band were detected in OE-12, OE-15, and OE-16 lines (Supplementary Figures 3C,D). The *ONAC096*-CP and *ONAC096*-OE plants showed no significant defect in growth and development, including plant height and panicle weight, in comparison to WT plants, when grown in greenhouse (Supplementary Figures 2D–I, 3E–I). The *onac096* mutant line was identified by genotyping (Supplementary Figure 4A,B) and the *ONAC096* transcript in *onac096* plants was undetectable (Supplementary Figures 4C,D).

**FIGURE 3:**
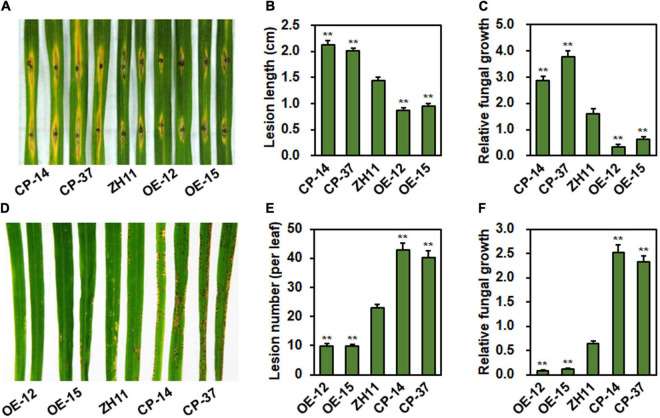
*ONAC096* positively regulates rice resistance against *M. oryzae*. **(A–C)** Disease symptom **(A)**, lesion length **(B),** and relative fungal growth **(C)** in the representative leaves of *ONAC096*-CP and *ONAC096*-OE plants. **(D–F)** Disease symptom **(D)**, lesion number **(E)**, and relative fungal growth **(F)** in the representative leaves of *ONAC096*-OE and *ONAC096*-CP plants. Detached leaves from 4-week-old plants were inoculated by dropping 5 μL spore suspension of *M. oryzae* (5 × 10^5^ spores/mL) **(A)** or 4-week-old plants were inoculated by foliar spraying of spore suspension of *M. oryzae* (2 × 10^5^ spores/mL) **(D)**. Images were taken and leaf samples were collected at 5 dpi. Relative fungal growth was presented as folds obtained by genomic qRT-RCR analyzing of the *M. oryzae MoPot2* gene level with the rice *OsUbq* gene level. Experiments were repeated at least three times with similar results, and results from one representative experiment are shown in panels **(A,D)**. Data presented (**B**,**C**,**E**,**F)** are the means ± SE from three independent experiments and asterisks indicate significant difference (*p* < 0.01, Student’s *t* test) in comparison to WT.

To explore the function of *ONAC096* in rice immunity against *M. oryzae*, resistance of the *ONAC096*-CP (CP-14 and CP-37) and *ONAC096*-OE (OE-12 and OE-15) lines was evaluated by inoculating with *M. oryzae* strain RB22 using punch inoculation and foliar spraying methods. In punch inoculation assays, lesions on detached leaves from *ONAC096*-CP14 and *ONAC096*-CP37 lines were larger, giving increases of 47 and 39.1% in lesion length, while lesions on leaves from *ONAC09*6-OE12 and *ONAC096*-OE15 lines were smaller, showing decreases of 39.9 and 33.9% in lesion length, as compared with those in WT plants (Figures 3A,B). The *ONAC096*-CP plants supported more *in planta* growth of *M. oryzae*, resulting in 79 and 135% increase over that in WT plants, while the *ONAC096*-OE plants supported less *in planta* growth of *M. oryzae*, leading to 80 and 92% decrease than that in WT plants (Figure 3C). In foliar spraying assays, the *ONAC096*-OE12 and *ONAC096*-OE15 plants showed smaller and thinner lesions with reduction of 57.1 and 56.9% in lesion numbers while *ONAC096*-CP14 and *ONAC096*-CP37 plants developed larger and denser lesions with an increase of 86.6 and 75.8% in lesion numbers, as compared to WT plants (Figures 3D,E). Accordingly, the *ONAC096*-OE plants supported less fungal growth, accounting for 12.5 and 17.2% of that in WT plants, while the *ONAC096*-CP plants provided more fungal growth, representing 394 and 363% of that in WT plants (Figure 3F). Punch inoculation assays revealed that lesions on detached leaves from *onac096* plants were larger (Supplementary Figure 4E), showing an increase of 80.2% in lesion length (Supplementary Figure 4F), and that the *onac096* plants supported more fungal growth, representing 3.63 folds of that in WT plants (Supplementary Figure 4G). Foliar spraying inoculation assays indicated that the *onac096* plants displayed more and larger disease lesions (Supplementary Figure 4H), giving an increase of 51.3% in lesion numbers (Supplementary Figure 4I), and supported a 2.74-fold higher level of fungal growth (Supplementary Figure 4J), as compared with WT plants. Collectively, these data indicate that overexpression of *ONAC096* enhanced while knockout of *ONAC096* attenuated rice resistance to blast, suggesting that *ONAC096* positively regulates rice immunity against *M. oryzae*.

**FIGURE 4:**
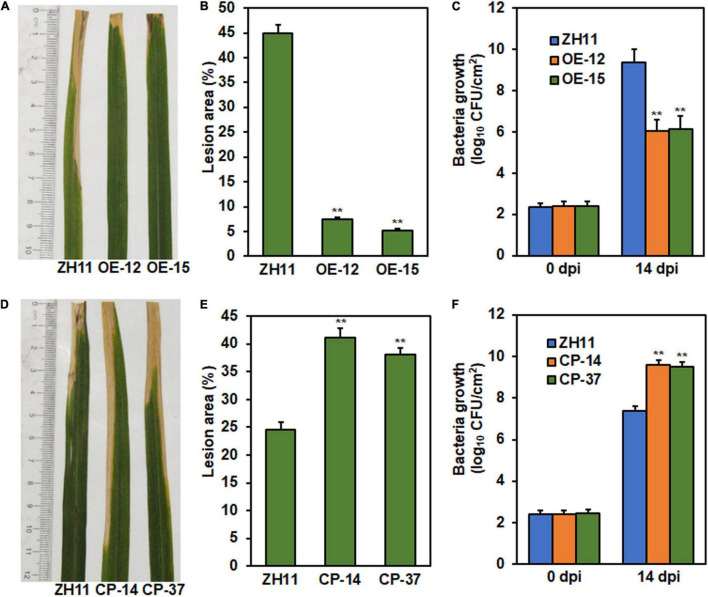
*ONAC096* positively regulates rice resistance against *Xoo.* **(A–C)** Disease phenotype **(A)**, lesion area **(B)**, and bacteria growth **(C)** in the representative leaves of *ONAC096*-OE plants. **(D–F)** Disease phenotype **(D)**, lesion area **(E)**, and bacteria growth **(F)** in the representative leaves of *ONAC096*-CP plants. Rice plants at booting stage were inoculated with bacterial suspension (OD600 = 0.8) by the leaf-clipping method **(A,D)**. Photographs were taken and leaf samples were harvested at 14 dpi. Bacterial growth was determined by counting the colony-forming units and presented as log_10_ (CFU/cm^2^) **(C,F)**. Experiments were repeated at least three times with similar results, and results from one representative experiment are shown in panels **(A,D)**. Data presented **(B**,**C**,**E,F)** are the means ± SE from three independent experiments and asterisks indicate significant difference (*p* < 0.01, Student’s *t* test) in comparison to WT.

To determine whether *ONAC096* has a function in rice immunity against other diseases, resistance of *ONAC096*-OE and *ONAC096*-CP lines at booting stage to *Xoo* was assessed by inoculating with *Xoo* strain PXO86 using leaf-clipping method. At 14 dpi, disease severity was lower in *ONAC096*-OE lines OE-12 and OE-15 plants (Figure 4A), leading to 83.5 and 88.5% of reduction in lesion areas (Figure 4B), while disease severity was higher in *ONAC096*-CP lines CP-14 and CP-37 plants (Figure 4D), resulting in 68 and 55.1% of increase in lesion areas (Figure 4E), as compared with that in WT plants. Similarly, bacterial growth in *ONAC096*-OE plants was markedly reduced by 35.5 and 34.6% while bacterial growth in *ONAC096*-CP plants was significantly increased by 30 and 29%, as compared with that in WT plants, at 14 dpi (Figures 4C,F). These results suggest that overexpression of *ONAC096* improved while knockout of *ONAC096* weakened rice resistance against bacterial leaf blight, indicating that *ONAC096* positively regulates rice immunity against *Xoo*.

### *ONAC096* Positively Regulates Pattern-Triggered Immunity in Rice

**FIGURE 5:**
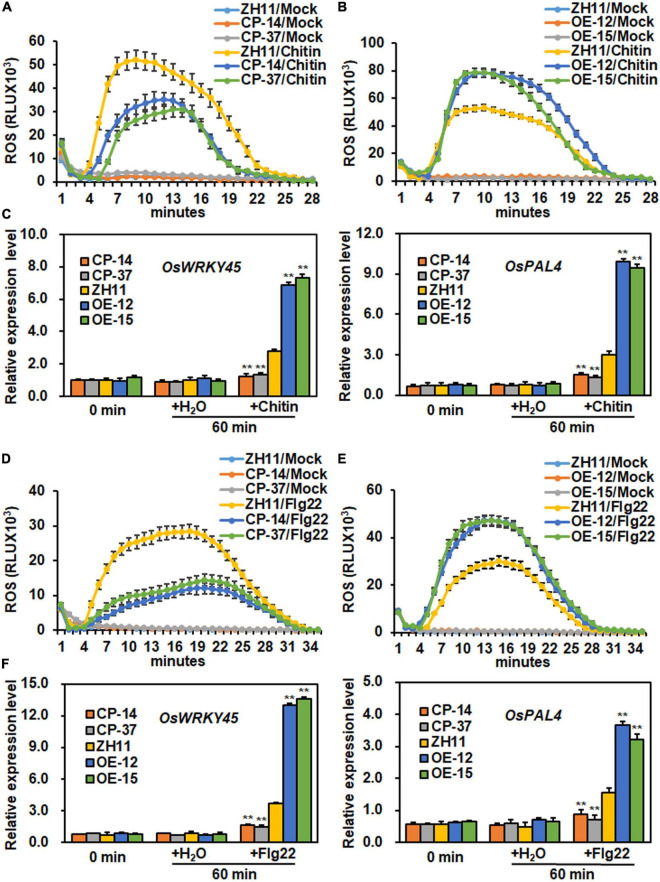
ONAC096 positively regulates chitin- and flg22-induced PTI in rice. **(A,B)** Chitin-induced ROS burst in *ONAC096*-CP plants **(A)** and *ONAC096*-OE plants **(B)**. **(C)** Expression changes of PTI marker genes in *ONAC096*-CP and *ONAC096*-OE plants after chitin treatment. **(D,E)** Flg22-induced ROS burst in *ONAC096*-CP plants **(D)** and *ONAC096*-OE plants **(E)**. **(F)** Expression changes of PTI marker genes in *ONAC096*-CP and *ONAC096*-OE plants after flg22 treatment. Leaf discs from 4-week-old plants were treated with 1 μM chitin, 100 nM flg22 or water and the chemiluminescent signal was immediately monitored at a 1 min interval over a period of 30 min. Results are presented as relative luminescence units (RLU) in panels **(A,B,D,E)**. Leaf samples were collected at 60 min after treatment with 10 nM chitin, 100 nM flg22 or water for analysis of gene expression. Relative expression of PTI marker genes were shown as folds of the expression level of the *18S rRNA* gene **(C,F)**. Experiments were repeated for three times with similar results. Data presented are the means ± SE from three independent experiments and asterisks **(C,F)** indicate significant difference (*p* < 0.01, Student’s *t* test) in comparison to WT.

To explore whether *ONAC096* is involved in PTI, the dynamics of chitin- or flg22-induced ROS burst and expression of PTI marker genes such as *OsWRKY45* and *OsPAL4* were analyzed and compared in *ONAC096*-CP, *ONAC096*-OE, and WT plants (Liu et al., 2017, 2019; Wang et al., 2019). In mock controls, no significant ROS burst and altered expression of *OsWRKY45* and *OsPAL4* were detected in leaves of *ONAC096*-OE, *ONAC096*-CP, and WT plants without chitin or flg22 treatment (Figure 5). After chitin treatment, ROS level in *ONAC096*-CP plants was ∼1.5 folds lower while ROS level in *ONAC096*-OE plants was nearly 1.5-fold higher than that in WT plants (Figures 5A,B). At 60 min after chitin treatment, lowered expression levels of *OsWRKY45* and *OsPAL4* with 54 and 53% reduction in leaves of *ONAC096*-CP plants while strengthened expression levels of *OsWRKY45* and *OsPAL14* with 150 and 220% increase in leaves of *ONAC096*-OE plants was detected, as compared with those in WT plants (Figure 5C). Similarly, ROS level in leaves of *ONAC096*-CP plants was reduced by ∼2.0 folds while ROS level in leaves of *ONAC096*-OE plants was increased by ∼1.6-fold, as compared to that in WT plants, after flg22 treatment (Figures 5D,E). At 60 min after flg22 treatment, the expression levels of *OsWRKY45* and *OsPAL4* in leaves of *ONAC096*-CP plants were significantly decreased by ∼42% and ∼52% while their expression levels in leaves of *ONAC096*-OE plants were dramatically strengthened by 360 and 330%, as compared with those in WT plants (Figure 5F). These results indicate that the knockout of *ONAC096* attenuated while overexpression of *ONAC096* strengthened ROS burst and expression of PTI marker genes in response to chitin and flg22, implying that ONAC096 positively regulates chitin- and flg22-induced PTI in rice.

### *ONAC096* Regulates the Expression of a Small Set of Defense and Signaling Genes

To elucidate the molecular mechanism of *ONAC096* in rice immunity and identify its target genes, RNA-seq transcriptome profiling of mock- or *M. oryzae*-inoculated *ONAC096*-CP, *ONAC096*-OE, and WT plants was performed. Because ONAC096 is a transcriptional activator (Figure 2) that positively regulates rice immunity (Figures 3–5), genes up-regulated in *ONAC096*-OE and down-regulated in *ONAC096*-CP plants were concerned. With criteria of expression change ≥ 1.5 folds and *P* < 0.05, a total of 77 genes were found to be up-regulated in *ONAC096*-OE and down-regulated in *ONAC096*-CP plants (Supplementary Figure 5A and Supplementary Table 3), as compared with WT plants, without *M. oryzae* infection. At 24 h after *M. oryzae* inoculation, 41 genes were up-regulated in *ONAC096*-OE but down-regulated in *ONAC096*-CP plants, as compared with those in infected WT plants (Supplementary Figure 5A and Supplementary Table 4). Collectively, 28 genes were found to be up-regulated in mock-inoculated and *M. oryzae*-infected *ONAC096*-OE plants but down-regulated in mock-inoculated and *M. oryzae*-infected *ONAC096*-CP plants, in comparison to WT plants (Supplementary Figure 5A and Supplementary Table 2).

Differentially expressed genes (DEGs) in *ONAC096*-OE and *ONAC096*-CP plants after mock- or *M. oryzae*-inoculation were categorized into functional groups based on Gene Ontology (GO). DEGs after mock inoculation (Supplementary Table 3) or *M. oryzae* inoculation (Supplementary Table 4) were clustered into 24 (Supplementary Table 5) and 19 categories (Supplementary Table 6), respectively. The most overrepresented 13 categories include biological processes, cellular components, and molecular functions (Supplementary Figures 5B,C). Moreover, GO analysis of DEGs after mock- and *M. oryzae*-inoculation (Supplementary Table 2) revealed 19 categories of enriched genes (Supplementary Table 7), 13 of which were the most overrepresented categories (Supplementary Figure 5D).

**FIGURE 6:**
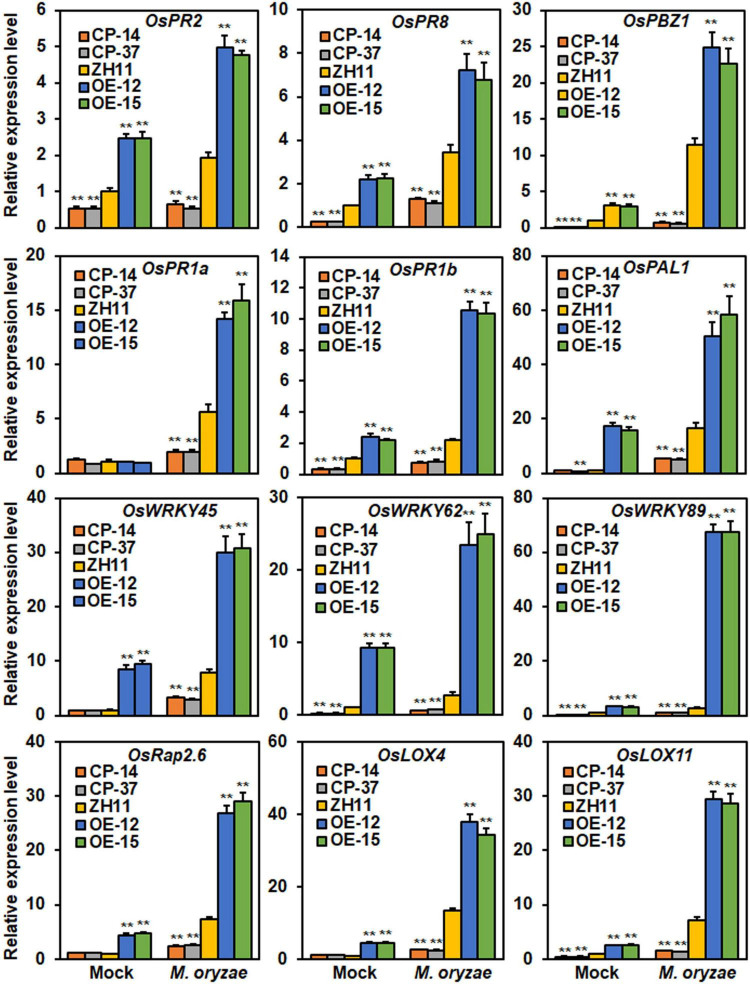
Differentially expression of selected defense and signaling genes in *ONAC096*-CP and *ONAC096*-OE plants with or without *M. oryzae* infection. Total RNA was extracted from *ONAC096*-CP, *ONAC096*-OE and WT plants after infection or non-infection of *M. oryzae*. Relative expression levels of the defense and signaling genes were normalized to the expression level of the *18s rRNA* gene. Experiments were repeated for three times with similar results. Data presented are the means ± SE from three independent experiments and asterisks indicate significant difference (*p* < 0.01, Student’s *t* test) in comparison to WT.

Among the DEGs (Supplementary Table 2), 12 genes have been previously reported to be involved in rice immunity, including five *PR* genes (*PR2*, *PR8*, *PR1a*, *PR1b*, and *PBZ1*), three *WRKY* genes (*WRKY45*, *WRKY62*, and *WRKY89*), two lipoxygenase genes (*LOX4* and *LOX11*), one phenylalanine ammonia-lyase (*PAL1*) and an ERF transcription factor (*Rap2.6*) (Wang et al., 2007; Marla and Singh, 2012; Wamaitha et al., 2012; Fukushima et al., 2016; Huang L. F. et al., 2016; Zhai et al., 2019). The expression of these genes in *ONAC096*-CP, *ONAC096*-OE, and WT plants after mock- or *M. oryzae*-inoculation was further validated by qRT-PCR. In mock-inoculated plants, the expression levels of the four *PR* genes (*OsPR2*, *OsPR8*, *OsPBZ1*, and *OsPR1b*), two *WRKY* genes (*OsWRKY62* and *OsWRKY89*), and one lipoxygenase gene *OsLOX11*, were markedly down-regulated in *ONAC096*-CP plants while significantly up-regulated in *ONAC096*-OE plants, as compared with those in WT plants (Figure 6). In addition, the expression levels of *OsWRKY45*, *OsLOX4*, *OsPAL1*, and *OsRap2.6* were up-regulated in *ONAC096*-OE plants; however, their expressions in *ONAC096*-CP plants were comparable to those in WT plants (Figure 6). In *M. oryzae*-inoculated plants, the expression levels of these genes were up-regulated in *ONAC096*-OE plants but down-regulated in *ONAC096*-CP plants, as compared with those in WT plants (Figure 6). These data consistently conformed the results from RNA-seq analysis and indicate that ONAC096 regulates a small set of defense and signaling genes that are involved in rice immunity.

### *ONAC096* Directly Activates the Expression of *OsRap2.6*, *OsWRKY62*, and *OsPAL1*

**FIGURE 7:**
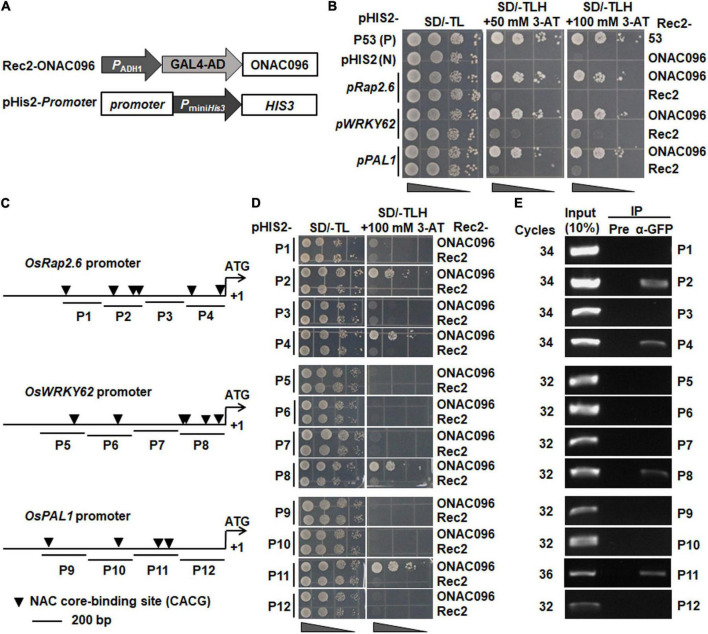
ONAC096 directly regulates the expression of *OsRap2.6*, *OsWRKY62*, and *OsPAL1*. **(A)** Schematic diagram of plasmids Rec2-ONAC096 and pHis2-*promoter* used in Y1H assay. **(B)** ONAC096 binds to the promoters of *OsRap2.6*, *OsWRKY62*, and *OsPAL1* in yeast cells. DNA-protein interactions were verified by growth performance of yeast cells co-transformed with indicated pairs of Rec2 and pHis2 vectors on SD/-Leu-/Trp/-His medium containing 50 mM or 100 mM 3-AT. **(C)** Diagram of putative NAC core-binding sites in the promoters of *OsRap2.6*, *OsWRKY62*, and *OsPAL1*. P1-P12 were the primers used in Y1H assays and probes used in ChIP-PCR assays. **(D)** Y1H assays of ONAC096 binding to the promoters of *OsRap2.6*, *OsWRKY62*, and *OsPAL1*. The partial truncated sequences of the promoters of *OsRap2.6*, *OsWRKY62*, and *OsPAL1* were fused into reporter vector pHis2, respectively. Transformants harboring each of the different truncated constructs with Rec2-ONAC096 or Rec2 empty vector (a negative control) were dropped by a series of 10-fold dilutions on plates of SD/-Trp-Leu, and SD/-Trp-Leu-His/100 mM 3-AT. **(E)** ChIP-PCR analysis of ONAC096 binding to the promoters of *OsRap2.6*, *OsWRKY62* and *OsPAL1*. ChIP of fragmented DNA isolated from *ONAC096*-GFP transgenic line incubated with GFP antibody (α-GFP) or pre-immune (Pre) serum (a negative control) and PCR analysis of IP samples and chromatin DNA before IP (a positive control/input) were performed using the P1-P12 primers. Experiments were repeated for three times with similar results, and results from one representative experiment are shown in panels **(B,D,E)**.

NAC TFs drive the expression of their downstream target genes through binding to the NAC core-binding sites (CACG) in the target gene promoters (Tran et al., 2004). To identify the direct target genes of ONAC096, Y1H assay was performed to examine the binding ability of ONAC096 to the promoters of these defense and signaling genes whose expression was up-regulated in *ONAC096*-OE but down-regulated in *ONAC096*-CP plants. To this end, the 1.5 Kb promoter regions from start codons of these genes were cloned into pHis2 vectors (Figure 7A) and co-transformed with expression construct Rec2-ONAC096 into yeast. All transformants grew well on SD/-Trp-Leu plates (Figure 7B). Transformants harboring Rec2-ONAC096 and pHis2-*pOsRap2.6*, pHis2-*pOsWRKY62*, or pHis2-*pOsPAL1* grew normally on the plates of SD/-Trp-Leu-His containing 50 mM 3-AT and SD/-Trp-Leu-His containing 100 mM 3-AT (Figure 7B). However, transformants carrying empty vector pGADT7-Rec2 with each of the pHis2-*promoter* vectors failed to grow on the same medium (Figure 7B), indicating that ONAC096 could directly bind to the promoters of *OsRap2.6*, *OsWRKY62*, and *OsPAL1*. By contrast, yeasts co-transformed with Rec2-ONAC096 and the rest of pHis2-*promoter* vectors grew normally on SD/-Trp-Leu medium but they did not grow on SD/-Trp-Leu-His medium with a high concentration of 3-AT (Supplementary Figure 6), suggesting that ONAC096 did not directly bind to the promoters of *OsPR2*, *OsPR8, OsPBZ1, OsPR1a, OsPR1b, OsWRKY45*, *OsWRKY89*, *OsLOX4*, and *OsLOX11*.

The 1.5 Kb promoter regions of *OsRap2.6*, *OsWRKY62*, and *OsPAL1* harbor six, six, and four NAC core-binding sites (CACG) (Tran et al., 2004), respectively (Figure 7C). Four probe regions for each promoter of *OsRap2.6*, *OsWRKY62*, and *OsPAL1* were chosen for Y1H and ChIP-PCR assays to map the binding sites of ONAC096 (Figure 7C). In Y1H assay, yeast cells co-transformed with different indicated pairs of pGADT7-Rec2 and pHis2 vectors grew well on SD/-Trp-Leu plates (Figure 7D). However, yeast cells co-transformed with Rec2-ONAC096 with pHis2-*pP2*, pHis2-*pP4*, pHis2-*pP8*, or pHis2*-pP11* grew on SD/-Trp/-Leu/-His medium containing 100 mM 3-AT (Figure 7D), indicating that ONAC096 could directly bind to the P2 and P4 regions in the *OsRap2.6* promoter, P8 region in the *OsWRKY62* promoter, and P11 region in the *OsPAL1* promoter. In ChIP-PCR assay, clear bands were observed in P2, P4, P8, and P11 probe regions; no band was visualized in P1, P3, P5, P6, P7, P9, P10, and P12 probe regions, when chromatin DNA was immunoprecipitated with anti-GFP antibody (Figure 7E). Together, these results revealed that ONAC096 bound to P2 (three NAC core-binding sites) and P4 (two NAC core-binding sites) regions in the *OsRap2.6* promoter, P8 region (four NAC core-binding sites) in the *OsWRKY62* promoter, and P11 region (two NAC core-binding sites) in the *OsPAL1* promoter.

**FIGURE 8:**
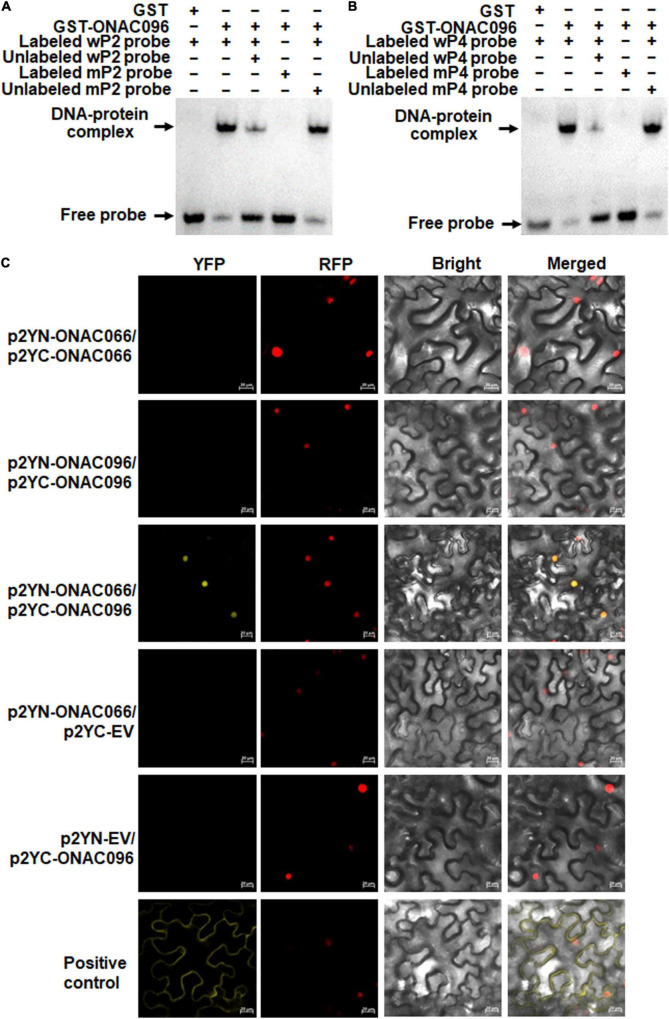
ONAC096 directly binds to the NAC core-binding sites in the *OsRap2.6* promoter and interacts with ONAC066. **(A,B)**, Binding of ONAC096 to the NAC core-binding sites in P2 **(A)** and P4 **(B)** regions of the *OsRap2.6* promoter. Biotin-labeled wP2/wP4 and mP2/mP4 probes (as the binding assay) or biotin-labeled wP2/wP4 probe in combination with excessive unlabeled wP2/wP4 or mP2/mP4 probe (as the competitive assay) were incubated with GST-ONAC096 or GST (a negative control). **(C)** OBAC096 interacts with ONAC066. Agrobacteria carrying indicated pairs of p2YC and p2YN plasmids were infiltrated into leaves of *N. benthamiana* plants expressing a red nuclear marker RFP-H2B protein and YFP signals were observed under a confocal laser scanning microscope at 48 h after infiltration. Images were taken in dark field for green fluorescence (*left*) and red fluorescence (*middle right*), white field for cell morphology (*middle left*) and in combination (*right*), respectively. Scale bar = 20 μm. Experiments were repeated for three times with similar results, and results from one representative experiment are shown.

The binding capacity of ONAC096 to the NAC core-binding sites in the *OsRap2.6* promoter was further confirmed by EMSA. As a result, ONAC096 bound to the biotin-labeled wP2 and wP4 fragments, forming specific DNA-protein complexes, but did not bind to the biotin-labeled mutated fragments mP2 or mP4 (Figures 8A,B). In competition binding assay, the binding capacity of ONAC096 to labeled wP2 and wP4 was attenuated by excessive unlabeled wP2 or wP4, but not affected by excessive unlabeled mP2 or mP4 (Figures 8A,B). These results indicate that ONAC096 specifically binds to the NAC core-binding sites in P2 and P4 regions in the *OsRap2.6* promoter.

### *ONAC096* Interacts With *ONAC066*

NAC TFs often exert their functions by forming homodimers or heterodimers (Walter et al., 2004; Olsen et al., 2005; Ren et al., 2021). The similar binding ability of ONAC096 and ONAC066 to the *OsWRKY62* promoter (Figure 7; Yuan et al., 2019b; Yuan X. et al., 2021) led to examine whether ONAC096 and ONAC066 could interact with each other. For this purpose, BiFC assay was conducted to examine the ONAC096-ONAC096, ONAC066-ONAC066, and ONAC096-ONAC066 interactions *in planta*. Accumulation of ONAC096 and ONAC066 proteins in *N. benthamiana* leaves co-infiltrated with agrobacteria harboring p2YN-ONAC066 and p2YC-ONAC066, p2YN-ONAC096 and p2YC-ONAC096, or p2YN-ONAC066 and p2YC-ONAC096 was comparable (Supplementary Figure 7). YFP signal was not detected in *N. benthamiana* leaves co-infiltrated with agrobacteria harboring p2YN-EV and p2YC-ONAC096, p2YN-ONAC066 and p2YC-EV, p2YN-ONAC066 and p2YC-ONAC066, or p2YN-ONAC096 and p2YC-ONAC096, while significant YFP fluorescence was clearly observed in leaves co-infiltrated with agrobacteria carrying p2YN-ONAC066 and p2YC-ONAC096 (Figure 8C). These results indicate that ONAC096 interacts with ONAC066.

## Discussion

Emerging evidence has indicated that the NAC TFs play significant roles in plant immunity (Ng et al., 2018; Yuan et al., 2019b; Bian et al., 2020). In rice, 9 NAC TFs have been demonstrated to be involved in rice immunity (Nakashima et al., 2007; Kaneda et al., 2009; Yoshii et al., 2009, 2010; Sun et al., 2013; Yokotani et al., 2014; Park et al., 2017; Liu et al., 2018; Wang et al., 2018; Yuan et al., 2019b; Yuan X. et al., 2021). The study revealed that *ONAC096*, which transcriptionally responded to *M. oryzae* and defense signaling hormones, positively regulates rice immunity against *M. oryzae* and *Xoo*, and demonstrated that ONAC096 directly binds to the promoters of *OsRap2.6, OsWRKY62* and *OsPAL1*. The study identifies a novel immunity-related rice NAC TF, ONAC096, and extends the importance of NAC TFs in rice immunity.

*ONAC096* is one of the 63 stress-related *ONAC* genes that exhibited overlapping expression patterns in rice stress response (Sun et al., 2015). Moreover, its Arabidopsis homologous *ANAC042* was reported to be involved in *Pseudomonas syringae*-induced defense response (Shahnejat-Bushehri et al., 2016) and its rice homologous *ONAC066* was found to strengthen resistance against *M. oryzae* and *Xoo* (Liu et al., 2018; Yuan et al., 2019b; Yuan X. et al., 2021). A recent study showed that the expression of *ONAC096* was up-regulated in response to ABA (Kang et al., 2019). In the present study, the expression of *ONAC096* was found to be significantly induced by *M. oryzae*, ABA and JA (Figure 1). This is also partially supported by the presence of several stress-related *cis*-elements including ABREs (Busk and Pagès, 1998) and CGTCA motifs (Rouster et al., 1997) in the *ONAC096* promoter, similar to *ONAC066* (Yuan et al., 2019a). Notably, the expression of *ONAC096* exhibited similar patterns during incompatible and compatible interactions between H8R/H8S and *M. oryzae* (Figure 1). ONAC096 can bind to a canonical NAC core binding sequence *NACRS* (Tran et al., 2004; Yuan et al., 2019b) and has transcription activator activity that is dependent on its C-terminal in yeast (Figure 2), which is similar to ONAC022 and ONAC066 reported in our previous studies (Hong et al., 2016; Yuan et al., 2019a). Collectively, ONAC096 should exert its roles in rice immunity by acting as a transcription activator to activate downstream target genes.

Phenotyping of the *ONAC096*-CP and *ONAC096*-OE plants as well as the *onac096* plants provided direct evidence suggesting an important role of *ONAC096* in rice immunity. The plant height and panicle weight for individuals of the *ONAC096*-CP and *ONAC096*-OE plants grown under greenhouse condition were comparable to WT (Supplementary Figures 2, 3), indicating that ONAC096 might be not involved in the growth and development processes. This is partially supported by the fact that no gene with known functions in rice growth and development appeared as differentially expressed genes, identified by RNA-seq analysis, in *ONAC096*-CP and *ONAC096*-OE plants without *M. oryzae* infection (Supplementary Table 3). Similarly, it was recently found that the *onac096* mutant exhibited similar fertility rate and panicle size (e.g., panicle length, grains/panicle, and 500-grain weight) to WT, but provided higher grain yield through increasing tiller number (Kang et al., 2019). Disease assays revealed that loss-of function of *ONAC096* in *ONAC096*-CP and *onac096* plants exhibited decreased while overexpression of *ONAC096* strengthened resistance to *M. oryzae* and *Xoo* (Figures 3, 4 and Supplementary Figure 4). PTI is the first layer of immune response in plants, which can be triggered via activation of PRRs by chitin, flagellin or other elicitor, accompanying a set of signaling cascades including ROS burst and induction of PTI-related defense genes (Wang et al., 2019; Kumar et al., 2021; Yuan et al., 2019b; Yuan X. et al., 2021). Overexpression of *ONAC096* triggered while knockout of *ONAC096* suppressed chitin- or flg22-induced ROS burst and PTI marker gene expression (Figure 5). Therefore, it is likely that ONAC096, acting as a positive regulator, is required for both PTI and resistance against *M. oryzae* and *Xoo* in rice. Whether ONAC096 plays a role in resistance against other pathogens needs to be investigated.

Among the NAC TFs involved in rice immunity, OsNAC6, OsNAC111, and ONAC066 function as transcription activators to drive their downstream target gene expression through directly binding to the promoters of these genes, conferring enhanced rice disease resistance (Nakashima et al., 2007; Yokotani et al., 2014; Liu et al., 2018; Yuan et al., 2019b; Yuan X. et al., 2021). As ONAC096 is a transcription activator (Figure 2), RNA-seq-based transcriptome profiling of mock- or *M. oryzae*-inoculated *ONAC096*-OE and *ONAC096*-CP plants identified 28 DEGs, which were up-regulated in *ONAC096*-OE plants and down-regulated in *ONAC096*-CP plants (Supplementary Figure 5 and Supplementary Table 2). Among the DEGs identified, 12 were defense and signaling genes that are involved in rice immunity (Supplementary Table 2). These genes were significantly up-regulated in *ONAC096*-OE plants but were remarkably suppressed in *ONAC096*-CP plants before and after *M. oryzae* infection (Figure 6). This is likely that ONAC096 regulates a specific small set of defense and signaling genes in rice. RNA-seq and qRT-PCR analysis revealed that expression of *OsPR2*, *OsPR8*, *OsPBZ1*, *OsPR1a*, *OsPR1b*, *OsWRKY45*, *OsWRKY89*, *OsLOX4*, and *OsLOX11* was significantly up-regulated in *ONAC096*-OE plants but markedly down-regulated in *ONAC096*-CP plants with or without *M. oryzae* infection (Figure 6 and Supplementary Table 2). However, Y1H assays revealed that ONAC096 did not bind to the promoters of these genes (Figure 7B and Supplementary Figure 6), indicating that the differentially expression of *OsPR2*, *OsPR8*, *OsPBZ1*, *OsPR1a*, *OsPR1b*, *OsWRKY45*, *OsWRKY89*, *OsLOX4*, and *OsLOX11* in *ONAC096*-OE and *ONAC096*-CP plants may be attributed indirectly to the ONAC096-activated defense signaling pathway. Notably, *OsPAL4* did not occur as a DEG in *ONAC096*-OE and *ONAC096*-CP plants with or without infection of *M. oryzae* in RNA-seq analysis. However, the expression of *OsPAL4* was significantly up-regulated in *ONAC096*-OE plants and down-regulated in *ONAC096*-CP plants after treatment of chitin or flg22 (Figure 5). This difference in expression changes of *OsPAL4* in *ONAC066*-OE and *ONAC096*-CP plants after *M. oryzae* infection or chitin and flg22 treatment might be due to different experimental procedures and techniques used.

Y1H, ChIP-PCR, and EMSA assays demonstrated that ONAC096 directly bound to the NAC core-binding sites in the promoters of *OsRap2.6*, *OsWRKY62*, and *OsPAL1* (Figures 7, 8). This is in line with a common knowledge that NAC TFs activate downstream target gene expression through binding to the NAC core-binding sites in the gene promoters (Tran et al., 2004; Yuan et al., 2019a,b; Yuan X. et al., 2021). These results indicate that *OsRap2.6*, *OsWRKY62*, and *OsPAL1* are targets of ONAC096 and ONAC096 modulates the transcription of *OsRap2.6*, *OsWRKY62*, and *OsPAL1* through directly binding to NAC core-binding sites in their promoters. *OsRap2.6*, encoding an ERF transcription factor, is a positive regulator that contributes to rice innate immunity through interacting with RACK1A in compatible interactions (Wamaitha et al., 2012). *PAL1* plays a critical role in biosynthesis of salicylic acid (SA) and the up-regulation of *PAL1* leads to enhanced plant defense against pathogen attack (Duan et al., 2014; Kim and Hwang, 2014). *OsPAL1* is rapidly induced by *M. oryzae* and is involved in rice blast resistance (Zhou et al., 2018; Zhai et al., 2019), implying that *OsPAL1* plays a positive role in rice immunity. It is likely that ONAC096 positively contributes to rice immunity through direct regulation of *OsRap2.6* and *OsPAL1*. However, the clear role of *OsPAL1* in rice immunity and involvement of *ONAC096* in SA-mediated defense response need further investigation.

ONAC096 and ONAC066 are phylogenetically closely related (Supplementary Figure 1) and both of them positively contribute to rice immunity against *M. oryzae* and *Xoo* (Figures 3, 4; Liu et al., 2018; Yuan et al., 2019b; Yuan X. et al., 2021). ONAC096 and ONAC066 function independently without functional redundancy in rice immunity as *ONAC096*-CP and *onac096* as well as *ONAC066*-RNAi and *onac066* plants exhibit clear phenotype in rice immunity and abiotic stress response (Figures 3, 4; Liu et al., 2018; Yuan et al., 2019a,b; Yuan X. et al., 2021). However, biochemical and molecular evidence suggests that ONAC096 and ONAC066 may coordinately function in rice immunity. ONAC096 interacted with ONAC066 *in planta* (Figure 8C). This is consistent with a general concept that the NAC TFs usually function as dimers (Olsen et al., 2005) and a specific example that ONAC127 and ONAC129 form heterodimers to regulate seed development and heat stress responses (Ren et al., 2021). Y1H and ChIP-PCR assays revealed that ONAC096 bound to P8 probe region in the *OsWRKY62* promoter (Figure 7), which is the same as that of ONAC066 (Yuan et al., 2019b; Yuan X. et al., 2021), indicating that ONAC096 and ONAC066 might act synergistically on the same NAC core-binding site in the *OsWRKY62* promoter. This suggests that *OsWRKY62* is the target gene for both ONAC096 and ONAC066. The ONAC096-ONAC066 interaction might result in the coordinate regulation of the expression of their common and specific target genes and/or the enhancement of binding activity to the promoters of downstream target genes. However, the effect of ONAC096-ONAC066 interaction on the binding activity of ONAC096 and ONAC066 and on the regulation of the expression of their target genes needs to be further investigated. The function of *OsWRKY62* in rice immunity have been inconsistently reported: *OsWRKY62* was shown to negatively regulate rice immunity (Peng et al., 2008, 2010; Liu et al., 2016; Liang et al., 2017) while it was also found to positively contribute to rice immunity against blast and bacterial leaf blight (Fukushima et al., 2016). It was suggested that OsWRKY45-OsWRKY62 heterodimers acts as a strong activator while the OsWRKY62 homodimers acts as a repressor of rice immunity (Fukushima et al., 2016). Surprisingly, expression of *OsWRKY45*, which is a positive regulator of rice immunity (Shimono et al., 2007, 2012; Goto et al., 2015, 2016) but is not a target of ONAC096 and ONAC066 (Supplementary Figure 6; Yuan et al., 2019b; Yuan X. et al., 2021), displays similar patterns with up-regulated expression in *ONAC096*-OE and *ONAC066*-OE plants and down-regulated expression in *ONAC096*-CP and *ONAC066*-RNAi plants (Figure 6; Yuan et al., 2019b; Yuan X. et al., 2021). Therefore, it is likely that the formation of the OsWRKY45-OsWRKY62 heterodimers, regulated directly and indirectly by ONAC096 and ONAC066, initiates the activation of defense response in rice upon pathogen infection.

Thousands of genes were expressed differentially in *M. oryzae*- and *Xoo*-infected rice (Kong et al., 2020), and ONACs play critical roles in transcriptional reprogramming of expression of genes for defense responses and different signaling pathways in rice response to *M. oryzae* and *Xoo*. Thus, it is likely that ONAC096, together with other immunity-related ONACs such as OsNAC6, OsNAC60, OsNAC111, ONAC066, ONAC122, and ONAC131 (Nakashima et al., 2007; Sun et al., 2013; Yokotani et al., 2014; Liu et al., 2018; Yuan et al., 2019b; Yuan X. et al., 2021), modulate the expression of downstream target genes and coordinately activate signaling pathways and defense response. On the other hand, SA, ABA, and JA are critical signaling hormones that regulate rice immunity (De Vleesschauwer et al., 2013; Yang et al., 2013). Some ONACs such as OsNAC60, ONAC066 and RIM1 play their functions in rice immunity through affecting SA, JA, and ABA signaling pathways (Yoshii et al., 2009, 2010; Liu et al., 2018; Wang et al., 2018). The responsiveness of *ONAC096* to exogenous MeJA and ABA (Figure 1) and the binding of ONAC096 to the *OsPAL1* promoter (Figure 7) may imply the requirement of SA, JA, and/or ABA signaling pathways in ONAC096-mediated immune response in rice upon pathogen infection. However, the relationship of ONAC096 with SA, JA, and/or ABA signaling pathways in rice immunity needs to be investigated.

## Conclusion

The present study demonstrated, through phenotyping of *ONAC096*-OE, *ONAC096*-CP and *onac096* lines, that ONAC096, as a pathogen- and defense hormone-inducible transcriptional activator, positively contributes to rice immunity against *M. oryzae* and *Xoo*. RNA-seq and biochemical analysis revealed that ONAC096 regulates a small set of defense and signaling genes and confirmed that ONAC096 directly targets to modulate the expression of *OsRap2.6*, *OsWRKY62*, and *OsPAL1*. Based on these data, a simple working model for the action of ONAC096 in rice immunity is proposed: Infection of pathogens, e.g., *M. oryzae*, induces the expression of *ONAC096*, and the ONAC096 protein targets to nuclei and binds to CACG elements in the promoters of defense and signaling genes including *OsRap2.6*, *OsWRKY62*, and *OsPAL1* to activate their expression, which initiates immune response against *M. oryzae* and *Xoo*. However, the detailed molecular network by which ONAC096 regulates rice immunity requires further investigation. Together with the observations that the *ONAC096*-OE plants did not show any significant defects on growth, development and yield, *ONAC096* is thus a promising gene that can be used in development of novel rice germplasm with improved broad-spectrum resistance against multiple fungal and bacterial diseases.

## Data Availability Statement

The datasets presented in this study can be found in online repositories. The names of the repository/repositories and accession number(s) can be found below: NCBI SRA under accession PRJNA776524 (https://www.ncbi.nlm.nih.gov/sra/PRJNA776524).

## Author Contributions

HW, DL, and FS conceived the project, designed the experiments, and analyzed the data. HW generated all material used in this study (cloning, vector, transformations, and transgenic plants). HW, YB, YG, YY, XY, XX, JW, and JL performed the experiments and collected the data. HW and FS drafted the manuscript. All authors commented on the manuscript.

## Conflict of Interest

The authors declare that the research was conducted in the absence of any commercial or financial relationships that could be construed as a potential conflict of interest.

## Publisher’s Note

All claims expressed in this article are solely those of the authors and do not necessarily represent those of their affiliated organizations, or those of the publisher, the editors and the reviewers. Any product that may be evaluated in this article, or claim that may be made by its manufacturer, is not guaranteed or endorsed by the publisher.
