# Supplementary material for: A Pathogen-Inducible Rice NAC Transcription Factor ONAC096 Contributes to Immunity Against Magnaprothe oryzae and Xanthomonas oryzae pv. oryzae by Direct Binding to the Promoters of OsRap2.6, OsWRKY62, and OsPAL1
Source: Front Plant Sci. 2021 Dec 10;12:802758. doi: 10.3389/fpls.2021.802758 (PMC8702954; doi:10.3389/fpls.2021.802758)
Supplement: Supplementary file 1 [file Data_Sheet_1.docx]

**SUPPLEMENTARY MATERIAL**

A Pathogen-inducible Rice NAC Transcription Factor ONAC096 Contributes to Immunity against *Magnaprothe oryzae* and *Xanthomonas oryzae* pv. *oryzae* by Direct Binding to Promoters of *OsRap2.6*, *OsWRKY62* and *OsPAL1*

***Hui Wang ^1^*, *Yan Bi ^1^*, *Yizhou Gao ^1^*, *Yuqing Yan ^1^*, *Xi Yuan ^2^*, *Xiaohui Xiong ^1^*, *Jiajing Wang ^1^*, *Jiayu Liang ^1^*, *Dayong Li ^1^* and *Fengming Song ^1^****

**
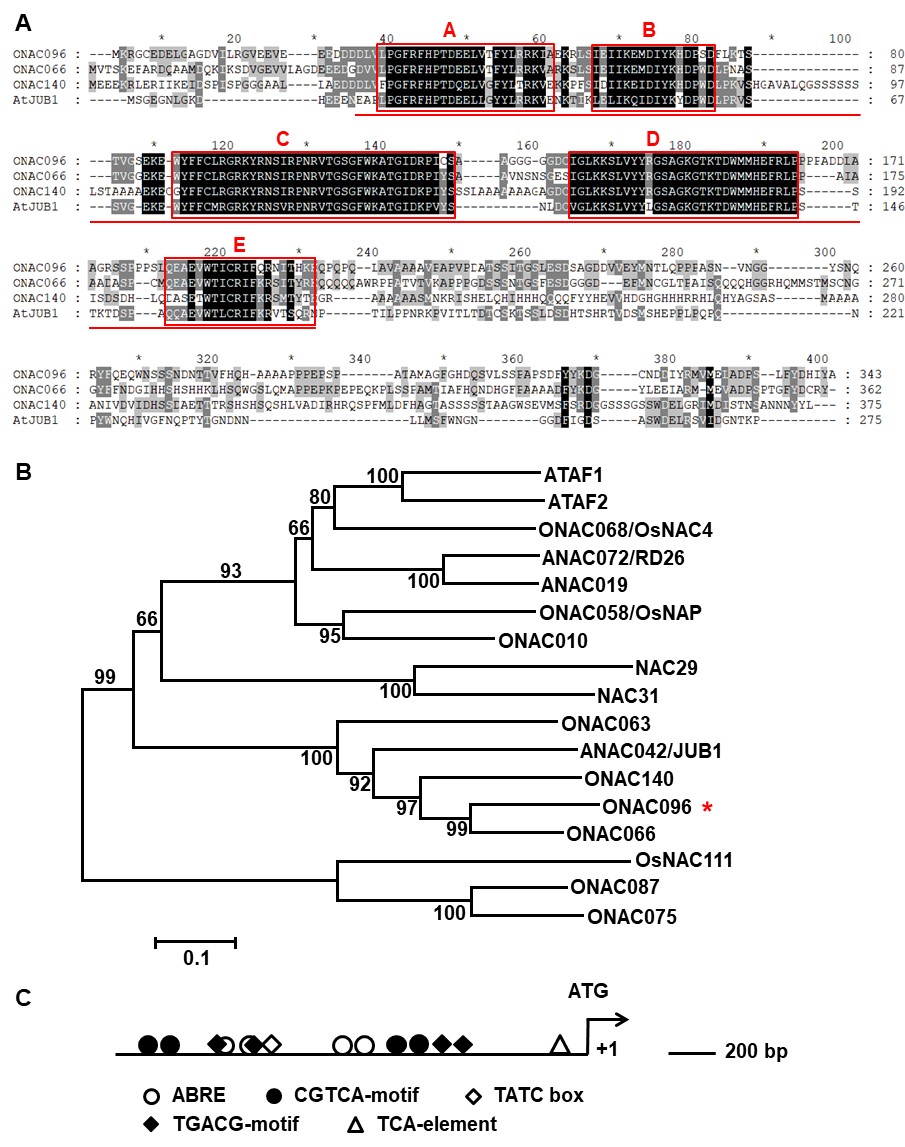
**

**Supplementary Figure S1 |** Sequence alignment and phylogenetic tree analysis of ONAC096 and *cis*-elements in promoter of ONAC096. **(A)** Alignment of ONAC096 with rice ONAC066, ONAC140 and Arabidopsis AtJUB1. Identical amino acids are shaded in black and the conserved NAC domain is underlined in red while the five highly conserved subdomains A to E are boxed with red lines. **(B)** Phylogenetic tree analysis of ONAC096 with other known stress-responsive NAC proteins. Multiple alignments were performed using Clustal X2 program and phylogenic tree was constructed by Neighbour joining method using MEGA7.0. Proteins used and their accessions are as follow: Arabidopsis ATAF1 (At1g01720), ATAF2 (At5g08790), ANAC019 (At1g52890), ANAC042/AtJUB1 (At2g43000), ANAC072/RD26 (At4g27410), rice ONAC066 (Os03g56580), ONAC096 (Os07g04560), ONAC140 (Os12g43530), ONAC063 (Os08g33910), ONAC068/OsNAC4 (Os01g60020), ONAC075 (Os01g66490), ONAC087 (Os05g34600), ONAC017/OsNAC111 (Os11g05614), ONAC058/OsNAP (Os03g21060) and ONAC010 (Os07g37920). **(C)** Distribution of major stress-related *cis*-elements in the promoter region (1.5 Kb upstream of ATG) of the *ONAC096* gene.

**
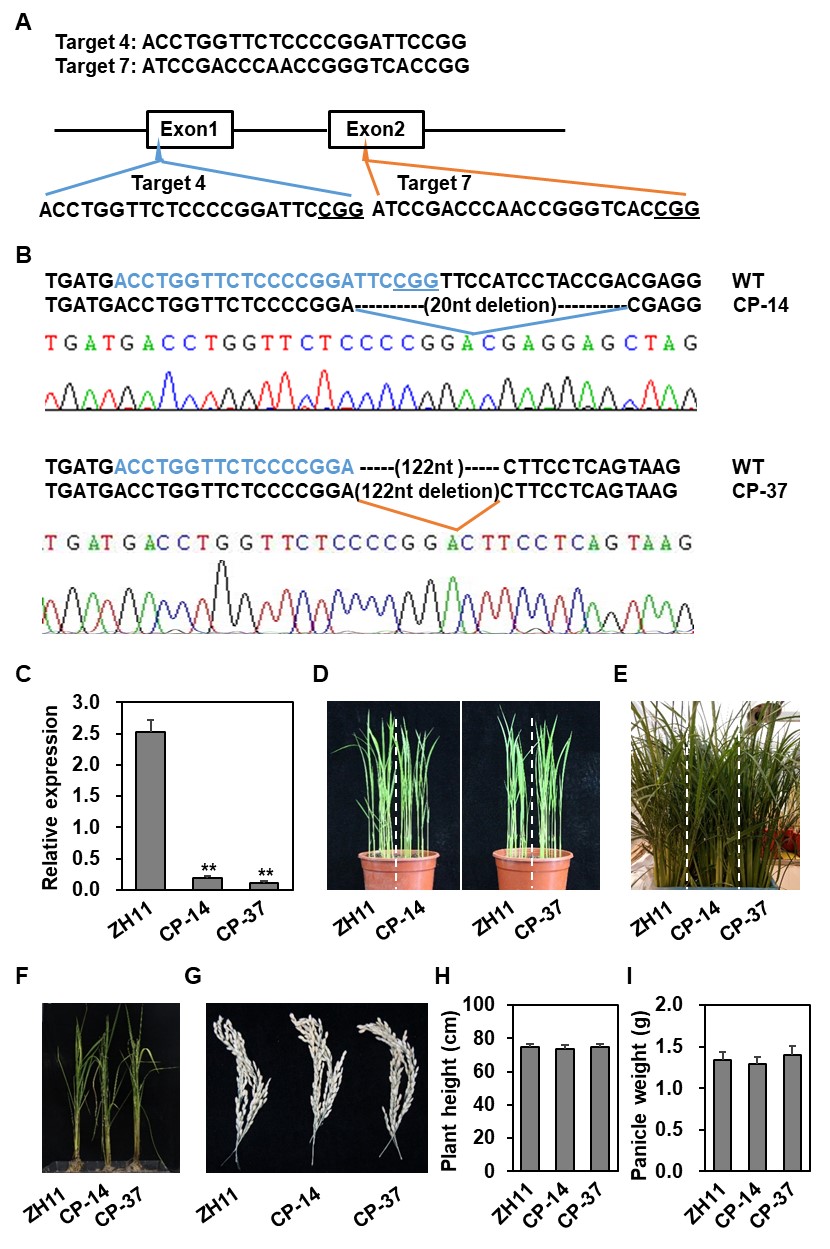
**

**Supplementary Figure S2 |** Molecular characterization and growth performance of *ONAC096*-CP lines. **(A)** Target sequence and its position in *ONAC096* gene. **(B)** Sequence alignment of *ONAC096* gene between *ONAC096*-CP lines and WT plants. WT, wild-type plants. CP-14 and CP-37, two lines of *ONAC096*-CRISPR/Cas9 plants. **(C)** Transcript level of *ONAC096* in *ONAC096*-CP lines. **(D-F)** Growth phenotype of the *ONAC096*-CP plants at two-week-old seedling stage **(D)**, at tiller stage **(E)** and at heading stage **(F)** grown in greenhouse. **(G)** Panicles from the *ONAC096*-CP and WT plants grown in greenhouse. **(H-I)** Plant height **(H)** and panicle weight **(I)** of the *ONAC096*-CP and WT plants grown in greenhouse. Data presented **(C, H and I)** are the means ± SE from three independent experiments. Asterisks in **(C)** indicate significant difference (*p*<0.01, Student’s *t*-test) and no statistically significant difference (*p* = 0.05, Student’s *t*-test) was detected in panels **(H** **and I)** according to WT.

**
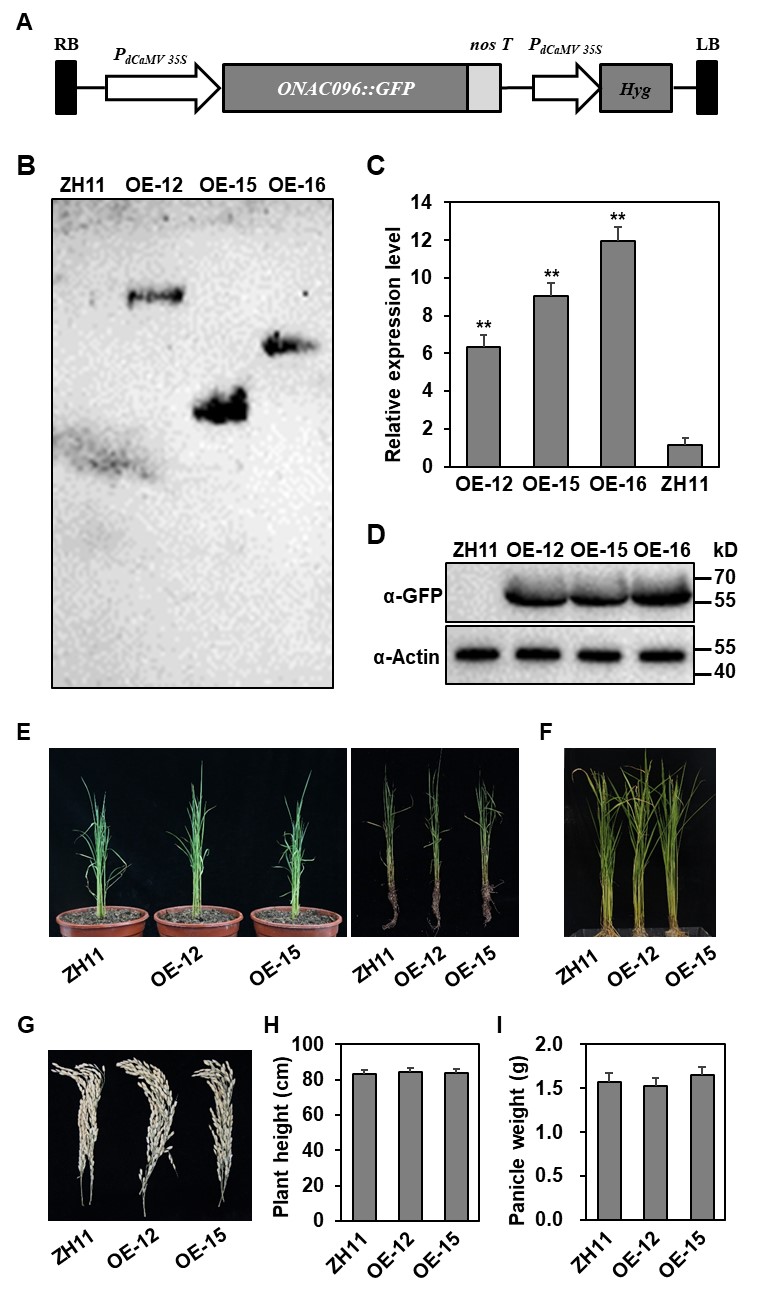
**

**Supplementary Figure S3 |** Molecular characterization and growth performance of *ONAC096*-OE lines. **(A)** Schematic diagram of the *ONAC096* overexpression construct. **(B)** Southern blot analysis to confirm the copy number in *ONAC096*-OE transgenic lines. **(C)** Transcript level of *ONAC096* in *ONAC096*-OE lines. **(D)** Western blot analysis to detect the ONAC096-GFP protein in *ONAC096*-OE lines. **(E-F)** Growth phenotype of the *ONAC096*-OE plants at four-week-old seedling stage **(E)** and at heading stage **(F)** grown in greenhouse. **(G)** Panicles from the *ONAC096*-OE and WT plants grown in greenhouse. **(H-I)** Plant height **(H)** and panicle weight **(I)** of the *ONAC096*-OE and WT plants grown in greenhouse. Experiments in **(B, C and D)** were repeated for three times with similar results, and results from one representative experiment are shown in panels **(B and D)**. Data presented **(C**, **H and** **I)** are the means ± SE from three independent experiments. Asterisks in **(C)** indicate significant difference (*p*<0.01, Student’s *t*-test) and no statistically significant difference (*p* = 0.05, Student’s *t*-test) was detected in panels **(H** **and I)** according to WT.

**
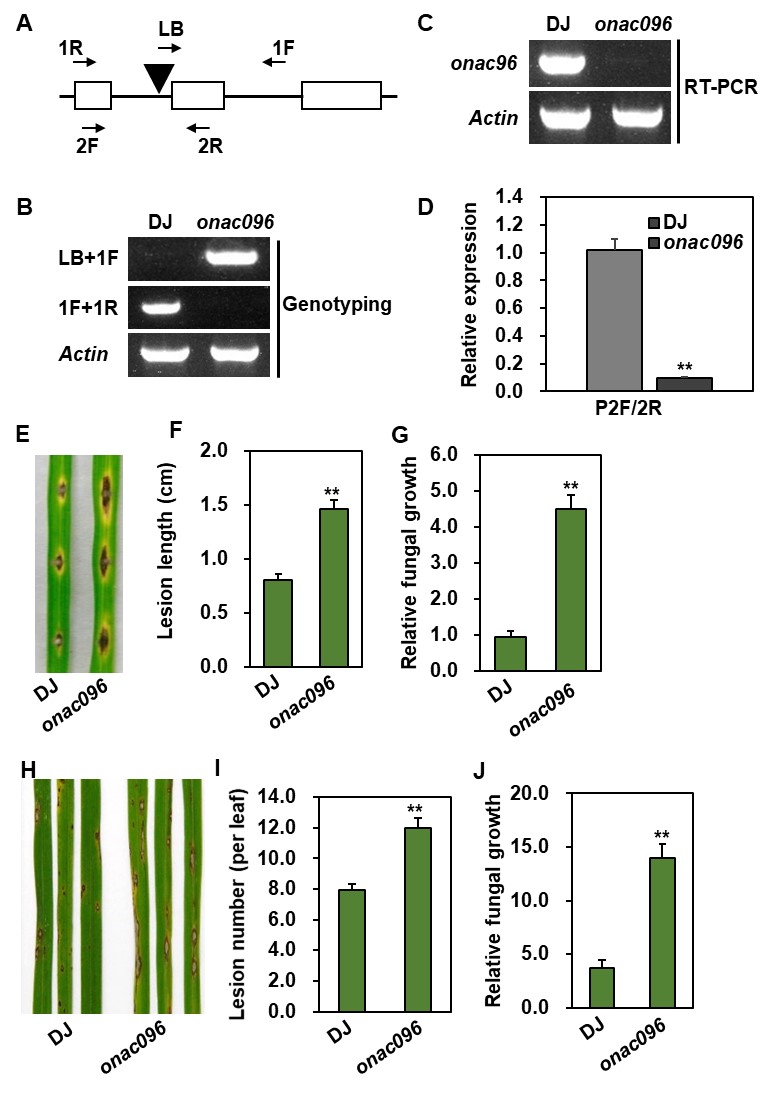
**

**Supplementary Figure S4 |** The *onac096* mutant showed reduced resistance against *Magnaporthe oryzae*. **(A)** *ONAC096* gene structure and T-DNA location in *onac096* mutant. Open boxes indicate the exons while black lines indicate introns. Filled triangle indicates the location T-DNA in *onac096* mutant. Primers used for genotyping, RT-PCR and qRT-PCR analyses are indicated. **(B)** Genotyping of *onac096* mutant using genomic DNA PCR analysis. 1F and 1R were used as gene-specific primers and 2715LB was a T-DNA-specific primer. **(C-D)** Detection of *ONAC096* transcript in *onac096* mutant using RT-PCR **(C)** and qRT-PCR analysis **(D)**. 2F and 2R were used as qRT-PCR primers. DJ, Dongjin WT plants. *onac096*, homozygous mutant plants. **(E-G)** Disease symptom **(E)**, lesion length **(F)** and relative fungal growth **(G)** in the representative leaves of *onac096* plants in detached leaf inoculation assays. **(H-J)** Disease symptom **(H)**, lesion number **(I)** and relative fungal growth **(J)** in the representative leaves of *onac096* plants in whole plant inoculation assays. Detached leaves from four-week-old plants were inoculated by dropping 5 μL spore suspension of *M. oryzae* (5×10^5^ spores/mL) **(E)** or four-week-old plants were inoculated by foliar spraying of spore suspension of *M. oryzae* (2×10^5^ spores/mlmL) **(H)**. Images were taken and leaf samples were collected at 5 dpi. Relative fungal growth was presented as folds obtained by genomic qRT-RCR analyzing of the *M. oryzae MoPot2* gene level with the rice *OsUbq* gene level. Experiments were repeated at least three times with similar results, and results from one representative experiment are shown in panels **(B, C, D, E and H)**. Data presented **(D, F, G, I and J)** are the means ± SE from three independent experiments and asterisks indicate significant difference (*p*<0.01, Student’s t test) in comparison to WT.

**
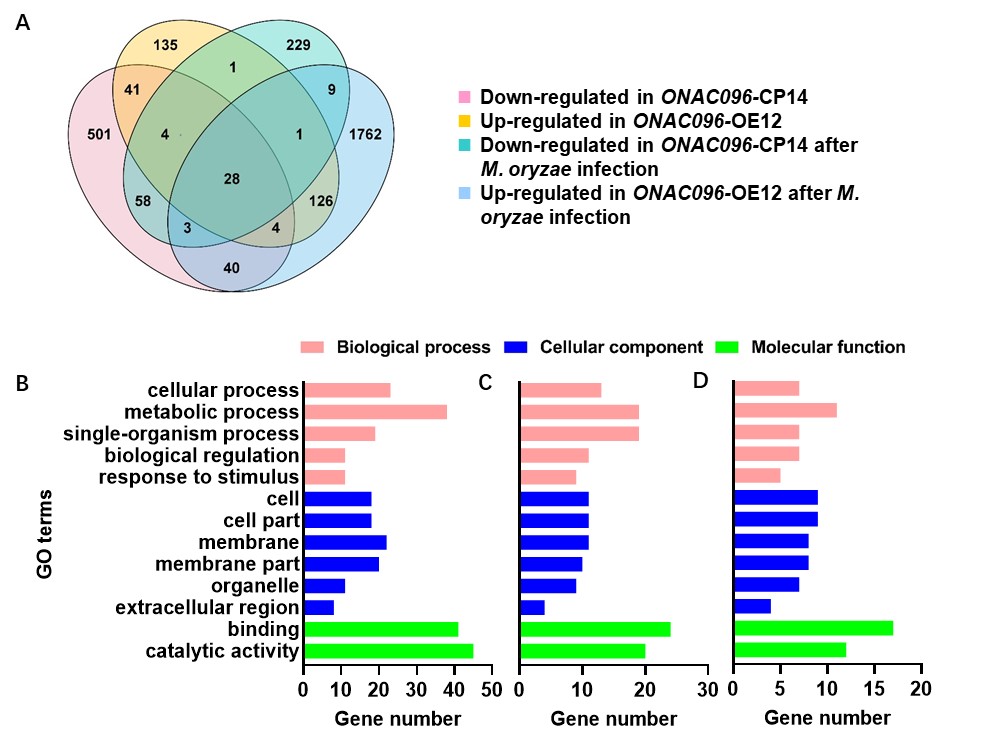
**

**Supplementary Figure S5 |** Differentially expressed genes in *ONAC096*-OE and *ONAC096*-CP plants. **(A)** Venn diagram showing differentially expressed genes down-regulated in *ONAC096*-CP plants and up-regulated in *ONAC096*-OE plants, in comparison to WT plants, with or without *M. oryzae* infection. The numbers in Venn diagram were obtained by comparing the gene expression levels in mock-inoculated *ONAC096*-OE/CP plants versus mock-inoculated WT plants and *M. oryzae*-inoculated *ONAC096*-OE/CP plants versus *M. oryzae*-inoculated WT plants. **(B-D)** Enriched GO terms of genes down-regulated in *ONAC096*-CP plants and up-regulated in *ONAC096*-OE plants, in comparison to WT plants, after mock inoculation **(B)** or *M. oryzae* inoculation **(C)** and mock- and *M. oryzae*-inoculation **(D)**.

**
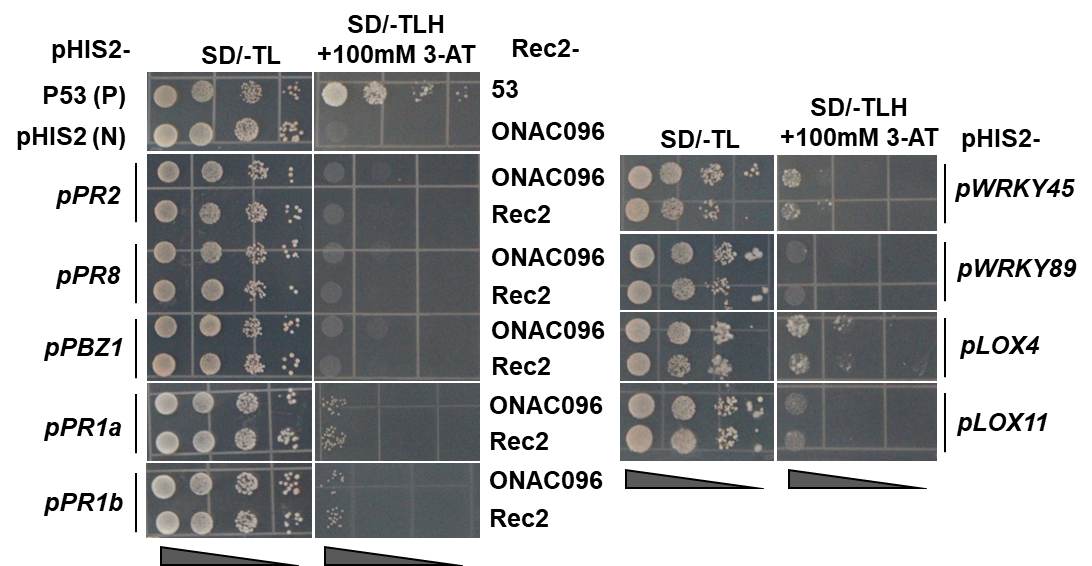
**

**Supplementary Figure S6 |** ONAC096 could not bind to the promoters of *OsPR2*, *OsPR8*, *OsPBZ1*, *OsPR1a*, *OsPR1b*, *OsWRKY45*, *OsWRKY89*, *OsLOX4* and *OsLOX11*. Growth performance of yeast transformants carrying each of the different reporter constructs pHis2-*promoter* with expression construct Rec2-ONAC096 or Rec2 empty vector (a negative control) on plates of SD/-Trp-Leu, and SD/-Trp-Leu-His/100 mM 3-AT. Experiments were repeated for three times with similar results and results from one representative experiment are shown.


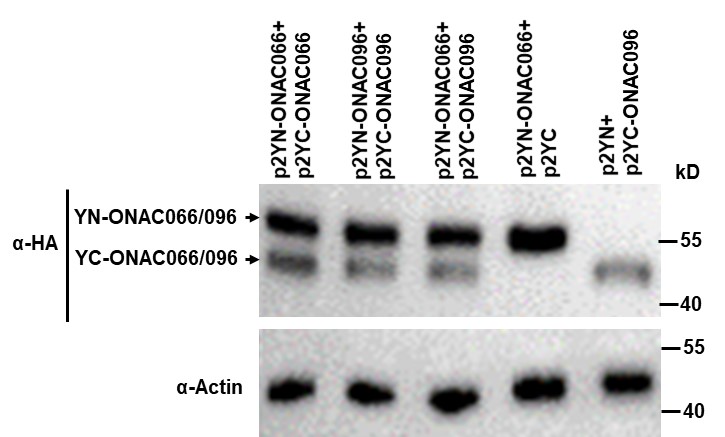


**Supplementary Figure S7 |** Detection of ONAC096 and ONAC066 accumulation in *N. benthamiana* leaves in BiFC assay. Agrobacteria carrying indicated pairs of p2YC and p2YN plasmids were infiltrated into leaves of *N. benthamiana* plants and leaf samples were collected at 48 h after infiltration. Fusion proteins were detected by HA antibody while equal loading of the samples was examined by Actin antibody.

**Supplemental Table S1 |** Primers used in this study.

| **Purpose** | **Primers** | **Sequences (5’-3’)** |
| --- | --- | --- |
| **Cloning** | *096*-F | ATGAAGAGGGGTTGTGAAGATGA |
|  | *096*-R | TTAAGCATATATATGATCATAGAACAAGCTC |
| **Subcellular localization** | *096*-GFP-F | AGCTTTCGCGAGCTCGGTACCATGAAGAGGGGTTGTGAA |
|  | *096*-GFP-R | GCCCTTGCTCACCATGGTACCAGCATATATATGATCATAGAACAAGC |
| **Identification of transgenic lines** | *096*-CP-F | TTGTGAAGATGAACTTGGAGCT |
|  | *096*-CP-R | GGCCTGGTCACTAAATCTGTCA |
|  | *096*-qRT-F | TCCAGAGGAACATCACCCACAAG |
|  | *096*-qRT-R | TGCTACTGTTCCATTGCTCCTG |
|  | *Hygromycin*-F | ACGGTGTCGTCCATCACAGTTTGCC |
|  | *Hygromycin*-R | TTCCGGAAGTGCTTGACATTGGGGA |
| **Genotyping of *onac096*** | 2715LB | ACGTCCGCAATGTGTTATTAA |
|  | *onac096*-LP | CACGTGTGAATGGAACCAAG |
|  | *onac096*-RP | TCCTACCGACGAGGAGCTAG |
|  | *onac096-*2F | TGAGCATCGAGATCATCAAGGAG |
|  | *onac96-*2R | GGGTCGGATGCTGTTCCTGTA |
| **Y1H** | BD-*096*-F1 | ATGGCCATGGAGGCCGAATTCATGAAGAGGGGTTGTGAAG |
|  | BD-*096*-R1 | CCGCTGCAGGTCGACGGATCCTTAAGCATATATATGATCATAGAACAA |
|  | BD-*096*-R2 | CCGCTGCAGGTCGACGGATCCGGCGGCAGAGCA |
|  | BD*-096*-F2 | ATGGCCATGGAGGCCGAATTCGCGGTGCCGGC |
|  | BD-*096*-R3 | CCGCTGCAGGTCGACGGATCCATGAAACACTGTGGTATTATCA |
|  | BD-*096*-F3 | ATGGCCATGGAGGCCGAATTCCAGCATGCGGCG |
|  | AD-*096*-F | GCCATGGAGGCCAGTGAATTCATGAAGAGGGGTTGTGAAG |
|  | AD-*096*-R | CAGCTCGAGCTCGATGGATCCTTAAGCATATATATGATCATAGAACAA |
|  | Rec2-*096*-F | GAGTGGCCATTATGGCCCATGAAGAGGGGTTGTGAAG |
|  | Rec2-*096-*R | GCCGACATGTTTTTTCCCTTAAGCATATATATGATCATAGAACAA |
|  | *NACRS-*F | AATTCCATGTGGAGCACGGAGCACGA |
|  | *NACRS-*R | CTAGTCGTGCTCCGTGCTCCACATGG |
|  | HIS2-*pPR2*-F | GACTCACTATAGGGCGAATTCAATCATAAGAAGAGGGAGTAGGGG |
|  | HIS2-*pPR2*-R | ATTACTAGTGGATCCACGCGTATGGCCTCTTGTCCTGCGA |
|  | HIS2-*pPR8*-F | GACTCACTATAGGGCGAATTCATAAAAATAAAGACTATTGAACGAAAAG |
|  | HIS2-*pPR8*-R | ATTACTAGTGGATCCACGCGTTGTTAATGTTAACTAGTAATTAAGCTTCG |
|  | HIS2-*pPBZ1*-F | GACTCACTATAGGGCGAATTCCACCGTCGTTGCGTTCG |
|  | HIS2-*pPBZ1*-R | ATTACTAGTGGATCCACGCGTCACTCAAGATATAATCTAACTAGCTAGCTAG |
|  | HIS2-*pPR1a*-F | GACTCACTATAGGGCGAATTCAATAATAAGTTCGGCCAATTT |
|  | HIS2-*pPR1a*-R | ATTACTAGTGGATCCACGCGTTAATTGAAGCTAGCTACTATATATATATATATA |
|  | HIS2-*pPR1b*F | GACTCACTATAGGGCGAATTCAATTCACTAGAAATTTTGGCATGAC |
|  | HIS2-*pPR1b*-R | ATTACTAGTGGATCCACGCGTAGTTAATCAAGCTCACACTTAATGTACG |
|  | HIS2-*pPAL1*-F | GACTCACTATAGGGCGAATTCCAAGGTTTTTATTTGTTGGTATATCAA |
|  | HIS2-*pPAL1*-R | ATTACTAGTGGATCCACGCGTTGCTACGGTGGTGGTGGTG |
|  | HIS2-*pW45*-F | GACTCACTATAGGGCGAATTCCTCAACAGTGACAACTAGATCCACA |
|  | HIS2-*pW45*-R | ATTACTAGTGGATCCACGCGTCCTCAATCCAAGCAAGCAAG |
|  | HIS2-*pW62*-F | GACTCACTATAGGGCGAATTCTTGGCAACAATGAGTTGTGGC |
|  | HIS2-*pW62*-R | ATTACTAGTGGATCCACGCGTGGCGGCAGCTAAGCTAGCTG |
|  | HIS2-*pW89*-F | GACTCACTATAGGGCGAATTCAGCAGCAGAATGGCTTGACTG |
|  | HIS2-*pW89*-R | ATTACTAGTGGATCCACGCGTCTTATGAGTTTGTCTATTATAGAGATGTGG |
|  | HIS2-*pRap2.6*-F | GACTCACTATAGGGCGAATTCATAAGTACATGTAAGCCCCTACTCATGAG |
|  | HIS2-*pRap2.6*-R | ATTACTAGTGGATCCACGCGTGGCGGAGGCGTCGTGG |
|  | HIS2-*pLOX4*-F | GACTCACTATAGGGCGAATTCAGCTGCACGGTGCACGC |
|  | HIS2-*pLOX4*-R | ATTACTAGTGGATCCACGCGTCTTCGCCTCTTCTGGATGTCTC |
|  | HIS2-*pLOX11*-F | GACTCACTATAGGGCGAATTCACATCTTTAATTTAGTCTCGGATTCAC |
|  | HIS2-*pLOX11*-R | ATTACTAGTGGATCCACGCGTTCTGACAAATTAAGTTGTCAGTGTGTC |
| **BiFC** | *096*-p2Y-F | CCCTTAATTAACATGAAGAGGGGTTGTGAAGATG |
|  | *096*-p2Y-R | GGGACTAGTAGCATATATATGATCATAGAACAAGCTCG |
|  | *066*-p2Y-F | CCCTTAATTAACATGGTGACCAGCAAGGAGTTTG |
|  | *066*-p2Y-R | GGGACTAGTATATCTACAGTCATAGAATCCTGTTGGACT |
| **qRT-PCR** | q*096*-qF | TGAAGAGGACTGGGAAGAGCTG |
|  | q*096*-qR | GCTATCTTGGACCATCGGTTGC |
|  | *MoPot-*F | ACGACCCGTCTTTACTTATTTGG |
|  | *MoPot*-R | AAGTAGCGTTGGTTTTGTTGGAT |
|  | *OsUbq*-F | TTCTGGTCCTTCCACTTTCAG |
|  | *OsUbq*-R | ACGATTGATTTAACCAGTCCATGA |
|  | q*18s*-F | ATGGTGGTGACGGGTGAC |
|  | q*18s*-R | CAGACACTAAAGCGCCCGGTA |
|  | q*PR2-*F | CCTTCACCAAGTATCTGCGA |
|  | q*PR2*-R | GTCTAGCGCATTCTGCAAAC |
|  | q*PR8-*F | TCTACGACGTGCAGAACAACTTCAG |
|  | q*PR8*-R | TCCAACTCAACCACTGTGCAAGTAA |
|  | q*PBZ1*-F | GTGGGAAGCACATACAAGACCC |
|  | q*PBZ1*-R | TCGTACTCCACCTTGAGCTTGG |
|  | q*PR1a*-F | TTATCCTGCTGCTTGCTGGTG |
|  | q*PR1a*-R | CCAGAAGATGTTCTCGCCGTAC |
|  | q*PR1b*-F | ACGGGCGTACGTACTGGCTA |
|  | q*PR1b*-R | CTCGGTATGGACCGTGAAG |
|  | q*PAL1*-F | ACCTGGTTCCCCTGTCCTACA |
|  | q*PAL1*-R | TTCATCACCTCGCAGAACACC |
|  | q*PAL4*-F | CTACCCGCTGATGAAGAAGC |
|  | q*PAL4*-R | GAACCTTGTTCAGCTCCTCG |
|  | q*WRKY45*-F | CAGGTGATGGAGGACATGGAGA |
|  | q*WRKY45*-R | GGTTCTTGACGACCACCGAAT |
|  | q*WRKY62*-F | GACAACCCCTACCCTAGAGCTTAC |
|  | q*WRKY62*-R | GTCGCCACTAGCATTGACCTATC |
|  | q*WRKY89*-F | GGACAACCACAACTTGGAGGAG |
|  | q*WRKY89*-R | TCATTGTGAGGTGCTTGAGCC |
|  | q*Rap2.6*-F | AGCAGGCTTCTTCCTTCTACCC |
|  | q*Rap2.6*-R | CTTCTTCGGGTCCCTTATCTCC |
|  | q*LOX4*-F | GTTCGACTCGTTCCAGGACATC |
|  | q*LOX4*-R | CGCCTCCTTGTCCTGTTTGATA |
|  | q*LOX11*-F | GCGGACATGGTTAATCGAGACT |
|  | q*LOX11*-R | CCAAGAGTTGCAGTGGAAAGTGA |
| **ChIP-PCR/Y1H** | P1-F | GACTCACTATAGGGCGAATTCAATCTCCTTGCTGGTAGTCTCAAGA |
|  | P1-R | ATTACTAGTGGATCCACGCGTTCTACTTTTGTGATAACACCTTCTCC |
|  | P2-F | GACTCACTATAGGGCGAATTCATTGAGATCCCTCTACCTTCTGTCTT |
|  | P2-R | ATTACTAGTGGATCCACGCGTTATATACTGGCTAGGCCACCGTG |
|  | P3-F | GACTCACTATAGGGCGAATTCAGTTGAATTGAAGCCTTGAAGGTATATAG |
|  | P3-R | ATTACTAGTGGATCCACGCGTTCTCCGGCCGCCGG |
|  | P4-F | GACTCACTATAGGGCGAATTCCTGGGGAGATCGATCGAGAGG |
|  | P4-R | ATTACTAGTGGATCCACGCGTGGCGGAGGCGTCGTGG |
|  | P5-F | GACTCACTATAGGGCGAATTCATTATAAATCATTCACTTATTTATTCATTCAGT |
|  | P5-R | ATTACTAGTGGATCCACGCGTCTAAGAAGGTTAAGGTGATGTGGATG |
|  | P6-F | GACTCACTATAGGGCGAATTCATTTACCGCTCTAACATCCCACATA |
|  | P6-R | ATTACTAGTGGATCCACGCGTTTATCTTTAGATACGTAGTCAGGATTTGC |
|  | P7-F | GACTCACTATAGGGCGAATTCTACATGCCTAAATATATAGCAAGGATTTG |
|  | P7-R | ATTACTAGTGGATCCACGCGTGGCCTCGTAGGATATCAAAATTCTAC |
|  | P8-F | GACTCACTATAGGGCGAATTCATGAGTCACATGAGTAAGACGCTCG |
|  | P8-R | ATTACTAGTGGATCCACGCGTGGCGGCAGCTAAGCTAGCTG |
|  | P9-F | GACTCACTATAGGGCGAATTCATCTCCACATTCGTTTGTTTTGTAA |
|  | P9-R | ATTACTAGTGGATCCACGCGTGGCTGGACTGTCAAAATGGC |
|  | P10-F | GACTCACTATAGGGCGAATTCGTGTGCTGGCTCACACAGTCAG |
|  | P10-R | ATTACTAGTGGATCCACGCGTAAAAGGAAAACCAGGCTGCTCT |
|  | P11-F | GACTCACTATAGGGCGAATTCCAGGGTTGGTGAGATTTGTCC |
|  | P11-R | ATTACTAGTGGATCCACGCGTGTTTCTATATGGGTCGAACTATTTCTT |
|  | P12-F | GACTCACTATAGGGCGAATTCTAAGAGAAATTATGTGTTTCAAATATGGC |
|  | P12-R | ATTACTAGTGGATCCACGCGTTGCTACGGTGGTGGTGGTG |

**Supplementary Table S2 |** Differentially expressed genes down-regulated in *ONAC096*-CP plants and up-regulated in *ONAC096*-OE plants compared with the WT plants with or without infection of *Magnaporthe oryzae*.

| Gene ID | Description | Log_2_ FC  (Mock-CP/WT) | Log_2_ FC  (Mock-OE/WT) | Log_2_ FC  (*M. oryzae-*CP/WT) | Log_2_ FC  (*M. oryzae-*OE/WT) |
| --- | --- | --- | --- | --- | --- |
| LOC_Os07g04560 | ONAC096 | -3.80 | 9.75 | -3.91 | 8.21 |
| LOC_Os07g35560 | PR2/β-1,3-glucanase | -3.02 | 5.76 | -1.46 | 2.50 |
| LOC_Os01g47070 | PR8/chitinase III | -3.25 | 1.21 | -2.35 | 2.02 |
| LOC_Os07g03710 | PR1a | -2.42 | 6.21 | -1.97 | 3.28 |
| LOC_Os01g28450 | PR1b | -2.65 | 2.41 | -1.94 | 8.32 |
| LOC_Os12g36880 | PBZ1 | -3.23 | 0.83 | -4.13 | 1.70 |
| LOC_Os07g03730 | Similar to PR1a | -1.37 | 1.22 | -2.97 | 1.98 |
| LOC_Os05g25770 | WRKY45 | -1.47 | 3.10 | -2.54 | 1.78 |
| LOC_Os09g25070 | WRKY62 | -2.27 | 3.29 | -2.13 | 3.72 |
| LOC_Os11g02520 | WRKY89 | -1.13 | 1.84 | -1.62 | 5.92 |
| LOC_Os03g49350 | LOX4 | -1.11 | 2.17 | -4.23 | 1.71 |
| LOC_Os12g37260 | LOX11 | -1.18 | 1.03 | -1.74 | 2.21 |
| LOC_Os02g41630 | PAL1 | -2.51 | 4.05 | -1.36 | 4.08 |
| LOC_Os04g32620 | Rap2.6 | -1.48 | 2.02 | -1.45 | 1.94 |
| LOC_Os01g52790 | Cytochrome P450 | -0.88 | 0.73 | -2.21 | 0.90 |
| LOC_Os01g41810 | Cytochrome P450 | -1.05 | 0.69 | -3.11 | 1.15 |
| LOC_Os07g23570 | Cytochrome P450 | -2.72 | 0.87 | -1.86 | 5.03 |
| LOC_Os09g26780 | JAZ8 | -3.70 | 0.99 | -2.63 | 3.20 |
| LOC_Os03g08320 | JAZ11 | -1.30 | 1.17 | -2.74 | 4.37 |
| LOC_Os01g65780 | Glycosyl transferase | -1.56 | 9.95 | -1.16 | 1.90 |
| LOC_Os12g14440 | Jacalin-related lectin | -1.04 | 4.07 | -2.02 | 3.87 |
| LOC_Os12g29690 | NBS-LRR protein | -3.00 | 12.04 | -1.99 | 11.74 |
| LOC_Os04g20680 | WAK3 | -1.02 | 2.32 | -2.71 | 1.99 |
| LOC_Os01g68870 | LRR-RLK | -0.80 | 1.60 | -2.30 | 2.24 |
| LOC_Os09g29510 | WAK protein | -1.19 | 2.10 | -3.55 | 1.83 |
| LOC112936121 | Receptor-like protein | -3.11 | 0.98 | -3.65 | 1.76 |
| LOC4324405 | Receptor like protein | -1.12 | 0.96 | -2.30 | 1.84 |
| LOC4351960 | Pik-2-like | -1.00 | 1.32 | -1.95 | 4.49 |
| Os10g0469300 | LRR protein | -1.33 | 2.01 | -4.13 | 2.35 |

*P*-value for the expression changes of all genes listed is <0.01.

**Supplementary Table S3 |** Differentially expressed genes down-regulated in *ONAC096*-CP plants and up-regulated in *ONAC096*-OE plants compared with the WT plants without *M. oryzae* infection.

| Gene ID | Description | Log_2_FC  (Mock-CP/WT) | Log_2_FC  (Mock-OE/WT) |
| --- | --- | --- | --- |
| LOC_Os07g35560 | PR2/Beta-1,3-glucanase | -3.02 | 5.76 |
| LOC_Os01g47070 | PR8/chitinase III/OsChib3a | -3.25 | 1.21 |
| LOC_Os07g03710 | PR1a/Pathogenesis-related protein 1a | -2.42 | 6.21 |
| LOC_Os01g28450 | PR1b/Pathogenesis-related protein 1b | -2.65 | 2.41 |
| LOC_Os12g36880 | Pathogen resistance protein PBZ1 | -3.23 | 0.83 |
| LOC_Os05g25770 | OsWRKY45 | -1.47 | 3.10 |
| LOC_Os09g25070 | OsWRKY62 | -2.27 | 3.29 |
| LOC_Os11g02520 | OsWRKY89 | -1.13 | 1.84 |
| LOC_Os03g49350 | Lipoxygenase 4 | -1.11 | 2.17 |
| LOC_Os12g37260 | Lipoxygenase 11 | -1.18 | 1.03 |
| LOC_Os02g41630 | Phenylalanine ammonia-lyase 1 | -2.51 | 4.05 |
| LOC_Os02g41650 | Phenylalanine ammonia-lyase 2 | -1.75 | 6.13 |
| LOC_Os01g50720 | MYB family transcription factor | -1.73 | 6.40 |
| LOC_Os08g33940 | MYB family transcription factor | -1.99 | 1.77 |
| LOC_Os04g32620 | ERF transcription factor 101/OsRap2.6 | -1.48 | 2.02 |
| LOC_Os01g72370 | Ron-related bHLH transcription factor 2 | -0.81 | 0.76 |
| LOC_Os12g22120 | White-brown complex homolog protein 11 | -1.41 | 6.99 |
| LOC_Os12g22284 | White-brown complex homolog protein 11 | -2.29 | 9.78 |
| LOC_Os01g65780 | Glycosyl transferase | -1.56 | 9.95 |
| LOC_Os01g71680 | Glycosyl hydrolases family 17 | -1.48 | 2.93 |
| LOC_Os01g71340 | Glycosyl hydrolases family 17 | -2.22 | 4.37 |
| LOC_Os01g71350 | Glycosyl hydrolases family 17 | -2,12 | 2.38 |
| LOC_Os05g15770 | Glycosyl hydrolases family 18 | -1.08 | 4.20 |
| LOC_Os11g44950 | Glycosyl hydrolase family 3 protein | -2.90 | 1.06 |
| LOC_Os01g11620 | GDSL-like lipase/acylhydrolase | -2.85 | 3.26 |
| LOC_Os09g36880 | GDSL-like lipase/acylhydrolase | -1.76 | 1.30 |
| LOC_Os09g39430 | GDSL-like lipase/acylhydrolase | -2.29 | 1.06 |
| LOC_Os07g03730 | SCP-like extracellular protein | -1.37 | 1.22 |
| LOC_Os01g28500 | SCP-like extracellular protein | -2.31 | 1.38 |
| LOC_Os01g52790 | Cytochrome P450 | -0.88 | 0.73 |
| LOC_Os01g41810 | Cytochrome P450 | -1.05 | 0.69 |
| LOC_Os07g23570 | Cytochrome P450 | -2.72 | 0.87 |
| LOC_Os09g26780 | Jasmonate ZIM-domain protein 8 | -3.70 | 0.99 |
| LOC_Os03g08320 | Jasmonate ZIM-domain protein 11 | -1.30 | 1.17 |
| LOC_Os01g52550 | ABC transporter, ATP-binding protein | -0.70 | 10.63 |
| LOC_Os01g68050 | Transmembrane amino acid transporter | -2.61 | 7.14 |
| LOC_Os11g02369 | Non-specific lipid-transfer protein 2 | -4.09 | 1.37 |
| LOC_Os01g66860 | Serine/threonine protein kinase | -3.04 | 8.52 |
| LOC_Os11g01740 | Serine/threonine-protein kinase | -1.63 | 1.02 |
| LOC_Os10g39420 | Calcium-dependent protein kinase 3 | -0.85 | 0.76 |
| LOC_Os01g64490 | Protein kinase domain containing protein | -0.83 | 2.94 |
| LOC_Os11g35500 | Protein kinase protein | -1.87 | 2.54 |
| LOC_Os01g53240 | BURP domain containing protein | -2.46 | 8.50 |
| LOC_Os09g38440 | SET domain domain containing protein | -1.91 | 2.81 |
| LOC_Os01g67190 | Ribonuclease T2 family protein, | 1.87 | 4.73 |
| LOC_Os01g65550 | RNA recognition motif containing protein | -0.88 | 5.54 |
| LOC_Os11g11490 | Beta-lactamase-inhibitor protein II | -0.89 | 1.23 |
| LOC_Os02g05900 | Unknown function DUF547 protein | -0.87 | 1.58 |
| LOC_Os12g29690 | NBS-LRR disease resistance protein | -3.00 | 12.04 |
| LOC4351960 | Disease resistance protein Pik-2-like | -1.00 | 1.32 |
| LOC107279290 | Disease resistance protein RGA5-like | -4.93 | 2.57 |
| LOC_Os06g42800 | Strubbelig-receptor family 8 precursor | -2.44 | 2.09 |
| LOC_Os04g20680 | Wall-associated receptor kinase 3 | -1.02 | 2.32 |
| LOC_Os01g68870 | Leucine-rich repeat receptor-like kinase | -0.80 | 1.60 |
| LOC_Os09g29510 | WAK receptor-like protein kinase | -1.19 | 2.10 |
| LOC112936121 | Receptor-like protein 7 | -3.11 | 0.98 |
| LOC4324405 | Receptor like protein 22-like | -1.12 | 0.96 |
| Os10g0469300 | Leucine-rich repeat protein | -1.33 | 2.01 |
| LOC_Os01g59819 | Os1bglu2-beta-glucosidase homologue | -3.89 | 2.89 |
| LOC_Os04g39864 | Os4bglu11-beta-glucosidase homologue | -1.94 | 0.68 |
| LOC_Os01g53294 | Respiratory burst oxidase homolog A | -0.71 | 11.56 |
| LOC_Os01g65690 | 4,5-DOPA dioxygenase extradiol | -6.73 | 3.05 |
| LOC_Os07g35940 | Beta-amylase | -3.77 | 1.33 |
| LOC_Os01g65790 | Pectinesterase | -1.36 | 0.93 |
| LOC_Os01g66710 | Polygalacturonase | -1.58 | 2.11 |
| LOC_Os12g24320 | ATPase 3 | -0.60 | 0.98 |
| LOC_Os12g25690 | UDP-glucose 6-dehydrogenase | -1.16 | 1.10 |
| LOC_Os07g48750 | Alpha-N-arabinofuranosidase | -1.29 | 1.47 |
| LOC_Os10g36848 | Coniferaldehyde 5-hydroxylase | -1.75 | 5.47 |
| LOC_Os01g54620 | Cellulose synthase catalytic subunit | -2.79 | 1.74 |
| LOC_Os06g12370 | OsFtsH6 FtsH protease | -0.92 | 1.00 |
| LOC_Os09g32080 | Membrane-associated chitinase | -1.52 | 2.67 |
| LOC_Os12g14440 | Mannose-binding jacalin-related lectin | -1.04 | 4.07 |
| LOC_Os01g32460 | Hypothetical protein | -2.56 | 1.12 |
| LOC_Os01g62980 | Hypothetical protein | -1.72 | 1.08 |
| LOC_Os01g66200 | Hypothetical protein | -2.63 | 5.57 |
| LOC_Os12g44230 | Hypothetical protein | -1.25 | 1.71 |

*P*-value for the expression changes of all genes listed is <0.01.

**Supplementary Table S4 |** Differentially expressed genes down-regulated in *ONAC096*-CP plants and up-regulated in *ONAC096*-OE plants compared with the WT plants after *M. oryzae* infection.

| Gene ID | Description | Log_2_FC  (*M. oryzae-*CP/WT) | Log_2_FC  (*M. oryzae-*OE/WT) |
| --- | --- | --- | --- |
| LOC_Os07g35560 | PR2/Beta-1,3-glucanase | -3.91 | 8.21 |
| LOC_Os01g47070 | PR8/chitinase III/OsChib3a | -1.46 | 2.50 |
| LOC_Os07g03710 | PR1a/Pathogenesis-related protein 1a | -2.35 | 2.02 |
| LOC_Os01g28450 | PR1b/Pathogenesis-related protein 1b | -1.97 | 3.28 |
| LOC_Os12g36880 | Pathogen resistance protein PBZ1 | -1.94 | 8.32 |
| LOC_Os07g03730 | SCP-like extracellular protein | -4.13 | 1.70 |
| LOC_Os05g25770 | OsWRKY45 | -2.97 | 1.98 |
| LOC_Os09g25070 | OsWRKY62 | -2.54 | 1.78 |
| LOC_Os11g02520 | OsWRKY89 | -2.13 | 3.72 |
| LOC_Os03g49350 | Lipoxygenase 4 | -1.62 | 5.92 |
| LOC_Os12g37260 | Lipoxygenase 11 | -4.23 | 1.71 |
| LOC_Os02g41630 | Phenylalanine ammonia-lyase 1 | -1.74 | 2.21 |
| LOC_Os04g32620 | ERF transcription factor 101/OsRap2.6 | -1.36 | 4.08 |
| LOC_Os11g37950 | WIP3-Wound-induced protein | -2.21 | 5.02 |
| LOC_Os11g37970 | WIP5-Wound-induced protein | -0.82 | 9.36 |
| LOC_Os01g52790 | Cytochrome P450 | -1.45 | 1.94 |
| LOC_Os01g41810 | Cytochrome P450 | -2.21 | 0.90 |
| LOC_Os07g23570 | Cytochrome P450 | -3.11 | 1.15 |
| LOC_Os09g26780 | Jasmonate ZIM-domain protein 8 | -1.86 | 5.03 |
| LOC_Os03g08320 | Jasmonate ZIM-domain protein 11 | -2.63 | 3.20 |
| LOC_Os01g65780 | Glycosyl transferase | -2.74 | 4.37 |
| LOC_Os03g61720 | Glycerol-3-phosphate acyltransferase | -3.08 | 1.68 |
| LOC_Os10g35070 | Alpha-galactosidase precursor | -0.60 | 0.69 |
| LOC_Os12g14440 | Mannose-binding jacalin-related lectin | -1.16 | 1.90 |
| LOC_Os02g43740 | Protein kinase KIPK | -1.96 | 1.91 |
| LOC_Os01g44110 | Serine/threonine-protein kinase | -2.52 | 1.42 |
| LOC_Os01g50400 | Serine/threonine protein kinase | -1.14 | 1.72 |
| LOC_Os12g31560 | Protein kinase protein | -2.00 | 3.99 |
| LOC_Os12g02200 | CBL-interacting protein kinase 14 | -0.68 | 1.50 |
| LOC_Os01g43910 | Mitogen-activated protein kinase 10 | -0.89 | 1.31 |
| LOC_Os12g29690 | NBS-LRR disease resistance protein | -2.02 | 3.87 |
| LOC_Os04g20680 | Wall-associated receptor kinase 3 | -1.99 | 11.74 |
| LOC_Os01g68870 | Leucine-rich repeat receptor-like kinase | -2.71 | 1.99 |
| LOC_Os09g29510 | WAK receptor-like protein kinase | -2.30 | 2.24 |
| LOC112936121 | Receptor-like protein 7 | -3.55 | 1.83 |
| LOC4324405 | Receptor like protein 22-like | -3.65 | 1.76 |
| LOC4351960 | Disease resistance protein Pik-2-like | -2.30 | 1.84 |
| Os10g0469300 | Leucine-rich repeat protein | -1.95 | 4.49 |
| LOC_Os12g13340 | Hypothetical protein | -0.83 | 0.88 |
| Oryza_sativa_newGene_3937 | -- | -0.60 | 1.64 |
| Oryza_sativa_newGene_6746 | -- | -1.38 | 0.64 |

*P*-value for the expression changes of all genes listed is <0.01.

**Supplementary Table S5 |** GO terms for differentially expressed genes down-regulated in *ONAC096*-CP plants and up-regulated in *ONAC096*-OE plants compared with the WT plants without *M. oryzae* infection.

| GO term | Description | Number in  input | Number in  background | *p*-value |
| --- | --- | --- | --- | --- |
| **Biological process** | | | | |
| GO:0009987 | cellular process | 23 | 8185 | 0.00158 |
| GO:0008152 | metabolic process | 38 | 7559 | 0.01196 |
| GO:0044699 | single-organism process | 19 | 5449 | 0.09409 |
| GO:0065007 | biological regulation | 11 | 2833 | 0.073117 |
| GO:0050896 | response to stimulus | 11 | 2431 | 0.058995 |
| GO:0071840 | cellular component organization/biogenesis | 2 | 1704 | 0.079845 |
| GO:0051179 | localization | 3 | 1354 | 0.099289 |
| GO:0023052 | signaling | 3 | 664 | 0.040725 |
| GO:0051704 | multi-organism process | 2 | 471 | 0.00968 |
| GO:0002376 | immune system process | 1 | 108 | 0.010286 |
| **Cellular component** | | | | |
| GO:0005623 | cell | 18 | 7888 | 0.029073 |
| GO:0044464 | cell part | 18 | 7888 | 0.029073 |
| GO:0016020 | membrane | 22 | 7134 | 0.002214 |
| GO:0044425 | membrane part | 20 | 6366 | 0.0299 |
| GO:0043226 | organelle | 11 | 6088 | 0.066746 |
| GO:0005576 | extracellular region | 8 | 782 | 0.035148 |
| **Molecular function** | | | | |
| GO:0005488 | binding | 41 | 11758 | 0.0265 |
| GO:0003824 | catalytic activity | 45 | 9709 | 0.049897 |
| GO:0005215 | transporter activity | 2 | 1061 | 0.061271 |
| GO:0001071 | nucleic acid binding activity | 5 | 721 | 0.024545 |
| GO:0016209 | antioxidant activity | 1 | 215 | 0.00978 |
| GO:0060089 | molecular transducer activity | 2 | 133 | 0.053226 |
| GO:0004871 | signal transducer activity | 2 | 133 | 0.053226 |
| GO:0000988 | transcription factor activity, protein binding | 2 | 96 | 0.0878 |

**Supplementary Table S6 |** GO terms for differentially expressed genes down-regulated in *ONAC096*-CP plants and up-regulated in *ONAC096*-OE plants compared with the WT plants after *M. oryzae* infection.

| GO term | Description | Number in  input | Number in  background | *p*-value |
| --- | --- | --- | --- | --- |
| **Biological process** | | | | |
| GO:0009987 | cellular process | 13 | 8185 | 0.00115 |
| GO:0008152 | metabolic process | 19 | 7559 | 0.01449 |
| GO:0044699 | single-organism process | 9 | 5449 | 0.094817 |
| GO:0065007 | biological regulation | 11 | 2833 | 0.06515 |
| GO:0050896 | response to stimulus | 9 | 2431 | 0.050425 |
| GO:0023052 | signaling | 3 | 664 | 0.034448 |
| GO:0051704 | multi-organism process | 3 | 471 | 0.005502 |
| **Cellular component** | | | | |
| GO:0005623 | cell | 11 | 7888 | 0.026939 |
| GO:0044464 | cell part | 11 | 7888 | 0.026939 |
| GO:0016020 | membrane | 11 | 7134 | 0.002551 |
| GO:0044425 | membrane part | 10 | 6366 | 0.028963 |
| GO:0043226 | organelle | 9 | 6088 | 0.065031 |
| GO:0005576 | extracellular region | 4 | 782 | 0.034312 |
| **Molecular function** | | | | |
| GO:0005488 | binding | 24 | 11758 | 0.023608 |
| GO:0003824 | catalytic activity | 20 | 9709 | 0.066795 |
| GO:0001071 | nucleic acid binding activity | 4 | 721 | 0.021989 |
| GO:0060089 | molecular transducer activity | 2 | 133 | 0.062021 |
| GO:0004871 | signal transducer activity | 2 | 133 | 0.062021 |
| GO:0000988 | transcription factor activity, protein binding | 2 | 96 | 0.087354 |

**Supplementary Table S7 |** GO terms for differentially expressed genes down-regulated in *ONAC096*-CP plants and up-regulated in *ONAC096*-OE plants compared with the WT plants with and without *M. oryzae* infection.

| GO term | Description | Number in  input | Number in  background | *p*-value |
| --- | --- | --- | --- | --- |
| **Biological process** | | | | |
| GO:0009987 | cellular process | 7 | 8185 | 0.00083 |
| GO:0008152 | metabolic process | 11 | 7559 | 0.012164 |
| GO:0044699 | single-organism process | 7 | 5449 | 0.094253 |
| GO:0065007 | biological regulation | 7 | 2833 | 0.065171 |
| GO:0050896 | response to stimulus | 5 | 2431 | 0.052549 |
| GO:0023052 | signaling | 1 | 664 | 0.037554 |
| GO:0051704 | multi-organism process | 1 | 471 | 0.008816 |
| **Cellular component** | | | | |
| GO:0005623 | cell | 9 | 7888 | 0.026939 |
| GO:0044464 | cell part | 9 | 7888 | 0.026939 |
| GO:0016020 | membrane | 8 | 7134 | 0.002551 |
| GO:0044425 | membrane part | 8 | 6366 | 0.028963 |
| GO:0043226 | organelle | 7 | 6088 | 0.065031 |
| GO:0005576 | extracellular region | 4 | 782 | 0.034312 |
| **Molecular function** | | | | |
| GO:0005488 | binding | 17 | 11758 | 0.025647 |
| GO:0003824 | catalytic activity | 12 | 9709 | 0.066795 |
| GO:0001071 | nucleic acid binding activity | 4 | 721 | 0.021639 |
| GO:0060089 | molecular transducer activity | 1 | 133 | 0.061864 |
| GO:0004871 | signal transducer activity | 1 | 133 | 0.061864 |
| GO:0000988 | transcription factor activity, protein binding | 2 | 96 | 0.087354 |
